# OTMODE: an optimal transport theory-based framework for identifying differential features in single-cell multi-omics data

**DOI:** 10.1093/bioinformatics/btaf650

**Published:** 2025-12-03

**Authors:** Huidong Su, Caicai Zhang, Frank Qingyun Wang, Chun Hing She, Xinxin Chen, Xiao Dang, Yao Lei, Ke Ni, Zewei Xiong, Danqing Yin, Xingtian Yang, Hong Feng, Philip H Li, Wanling Yang

**Affiliations:** Department of Paediatrics and Adolescent Medicine, LKS Faculty of Medicine, The University of Hong Kong, Hong Kong SAR, 999077, China; Department of Paediatrics and Adolescent Medicine, LKS Faculty of Medicine, The University of Hong Kong, Hong Kong SAR, 999077, China; Department of Paediatrics and Adolescent Medicine, LKS Faculty of Medicine, The University of Hong Kong, Hong Kong SAR, 999077, China; Department of Paediatrics and Adolescent Medicine, LKS Faculty of Medicine, The University of Hong Kong, Hong Kong SAR, 999077, China; Department of Paediatrics and Adolescent Medicine, LKS Faculty of Medicine, The University of Hong Kong, Hong Kong SAR, 999077, China; Department of Paediatrics and Adolescent Medicine, LKS Faculty of Medicine, The University of Hong Kong, Hong Kong SAR, 999077, China; Department of Paediatrics and Adolescent Medicine, LKS Faculty of Medicine, The University of Hong Kong, Hong Kong SAR, 999077, China; Computational Biology Department, Joint Carnegie Mellon–University of Pittsburgh Program in Computational Biology, Pittsburgh, PA, 15213, United States; Department of Psychiatry, Li Ka Shing Faculty of Medicine, The University of Hong Kong, Hong Kong SAR, 999077, China; Laboratory of Data Discovery for Health Limited (D24H), Hong Kong SAR, 999077, China; School of Biomedical Sciences, Li Ka Shing Faculty of Medicine, The University of Hong Kong, Pokfulam, Hong Kong SAR, 999077, China; Department of Paediatrics and Adolescent Medicine, LKS Faculty of Medicine, The University of Hong Kong, Hong Kong SAR, 999077, China; Department of Paediatrics and Adolescent Medicine, LKS Faculty of Medicine, The University of Hong Kong, Hong Kong SAR, 999077, China; Division of Rheumatology & Clinical Immunology, Department of Medicine, Queen Mary Hospital, The University of Hong Kong, Hong Kong SAR, 999077, China; Department of Paediatrics and Adolescent Medicine, LKS Faculty of Medicine, The University of Hong Kong, Hong Kong SAR, 999077, China

## Abstract

**Motivation:**

Single-cell technologies enable high-resolution cellular studies but face challenges in identifying differential features due to data complexity.

**Results:**

We present OTMODE, a non-parametric method using unbalanced Sinkhorn algorithm and Wald test to improve differential feature identification in single-cell multi-omics data. Under simulation, OTMODE achieved superior performance (average 90% *F*1 score; average 92% AUC score) with high efficiency (2.2 s for 5000 cells). In practice, it shows greater sensitivity than other state-of-the-art methods in detecting meaningful processes and can evaluate annotation accuracy by identifying potentially misannotated clusters from auto-annotation tools. Furthermore, OTMODE integrates seamlessly with Scanpy, offering a user-friendly solution for researchers.

**Availability and implementation:**

OTMODE is freely available at https://github.com/Eggong/OTMODE and also available at https://pypi.org/project/OTMODE/.

## 1 Introduction

Rapid development in single-cell omics technologies has provided unprecedented resolution on the perturbations induced by diseases and experimental manipulations. Such perturbations can lead to alternations of chromatin states and transcriptomic activities that can be detected by single-cell technologies. Specifically, single-cell RNA-seq (scRNA-seq) uncovers transcriptional heterogeneity within tissues, revealing distinct cellular states (Longo *et al.* 2021). Single-cell ATAC-seq (scATAC-seq) offers a unique window into chromatin accessibility, linking epigenetic regulation to transcriptional dynamics in individual cells (Buenrostro *et al.* 2015).

To detect these changes, conventional methods, such as *t*-test and Wilcoxon rank-sum test, suffer from reduced sensitivity because of the high sparsity of single-cell data (Stegle *et al.* 2015). To address the issues such as stochastic dropout and characteristic bimodal expression distributions, specialized methods, such as Monocle3 (Qiu *et al.* 2017) and MAST (Finak *et al.* 2015) have been developed to detect DEGs between conditions. However, these methods exhibit critical limitations: (1) bias toward highly expressed genes while being insensitive toward the genes with low expression levels (Soneson and Robinson 2018, Kharchenko 2021, Wu *et al.* 2025); and (2) poor interoperability with mainstream toolbox, such as Seurat (Hao *et al.* 2024) and Scanpy (Wolf *et al.* 2018). Additionally, the recent Memento approach demonstrates high sensitivity and computational efficiency on atlas-level datasets (Kim *et al.* 2024), however, its core Gaussian distribution assumption remains unvalidated for small datasets.

Pseudobulk approaches, such as DESeq2 (Love *et al.* 2014), circumvent sparsity via aggregating gene expression across cells within samples (Hafemeister and Halbritter 2023) but obscure cellular heterogeneity (Hu and Chikina 2024) and increases the analytical complexity. Models developed for transcriptomic data often perform suboptimally on scATAC-seq data due to fundamental differences in their underlying data distributions (Zhao *et al.* 2024). Collectively, these methodological gaps impede accurate identification of differential features and may delay biological discoveries.

Another critical challenge in differential feature identification is the absence of standardized pipelines for annotation (Heumos *et al.* 2023, Nouri *et al.* 2023). Single-cell annotation assigns cell types via cluster-specific differentially expressed genes (DEGs) and canonical cellular markers (Clarke *et al.* 2021). However, the complexity from high-dimensional data obscures distinctions between biologically meaningful clusters and technical artifacts (Vandenbon and Diez 2020). Although nowadays auto-annotation approaches, such as CellTypist (Domínguez Conde *et al.* 2022), have been developed, these approaches heavily depend on quality of reference databases and sometimes lead to annotation ambiguity (Pasquini *et al.* 2021), necessitating the involvement of expert supervision. This subjective reliance on researcher experiences risks downstream analytical validity. Thus, a quantitative metric is needed to assess annotation accuracy.

To address the above challenges, we introduce OTMODE, a Python-based framework leveraging optimal transport (OT) theory to detect differential features in single-cell data and to enhance annotation accuracy. OT theory provides a principled approach to compare probability distributions by finding the most efficient way to transform one distribution into another. This makes OT particularly well-suited for single-cell data analysis, as it naturally handles the inherent sparsity by focusing on the actual support of the distributions rather than requiring dense representations across the entire feature space. Furthermore, the powerful generalizability of OT allows it to easily scale to high-dimensional spaces without requiring dimension-specific assumptions (Altschuler *et al.* 2017, Genevay *et al.* 2018, Weed and Bach 2019), making it readily applicable to modern single-cell datasets that often encompass tens of thousands of features. Since OT’s geometric properties preserve the intrinsic structure of high-dimensional data, its ability to capture complex distribution differences make it ideal for the heterogeneous nature of single-cell measurements.

Here, we demonstrate the effectiveness of OTMODE in (1) sensitive and robust identification of differential features across conditions in single-cell multi-omics data; (2) improved annotation via a novel metric that quantifies the aggregate contribution of both positive and negative markers to each cell type; and (3) seamless integration with Scanpy and scverse ecosystem (Virshup *et al.* 2023) to facilitate downstream analysis. We validated OTMODE on both simulated and real scRNA-seq and scATAC-seq datasets (Supplementary Data 1, available as supplementary data at *Bioinformatics* online), demonstrating significant improvement of cell-type annotation and superior performance compared to state-of-the-art tools, including DESeq2, Memento, and SnapATAC2 (Supplementary Data 2, available as supplementary data at *Bioinformatics* online). Benchmarking against other OT-based methods also highlights its high computational efficiency.

## 2 Materials and methods

### 2.1 Methodology of OTMODE

OTMODE implements a comprehensive workflow for differential feature detection in single-cell data using OT theory. Our workflow begins by computing a cell-to-cell cost matrix using Euclidean distances between two groups of log10-normalized gene expression profiles. This matrix is then rescaled via min–max normalization to produce values between 0 and 1, thus rendering it suitable for subsequent OT calculations. The numbers of cells in Group 0 and Group 1 are determined to facilitate later distribution-based analyses. Principal component analysis (PCA) is then applied to both Group 0 and Group 1. The number of PCs for subsequent analysis are determined from elbow analysis. Following this dimensionality reduction, a Gaussian kernel density estimation is performed on each group’s principal component space to model the distribution of the transformed data. The default parameter of bandwidth (*h*) selection uses Scott’s rule (Scott 2015), which is a popular rule-of-thumb for automatically determining the bandwidth based on the variance and number of data points.

These density values are min–max normalized and serve as the empirical probability distributions (*μ* and *ν*) that represent each group’s underlying data distribution. Unlikely from other OT-based method (Nabavi *et al.* 2016), applying Wasserstein distance to detect differential features, OTMODE employed the Sinkhorn algorithm (Cuturi 2013), which computes the transport plan linking the two distributions by introducing a regularization parameter ($\lambda_{\mathrm{reg}}$). Also, a detailed theoretical background of Sinkhorn algorithm and a brief comparison between the two algorithms is provided in Supplementary Material Chapter 2, available as supplementary data at *Bioinformatics* online. Briefly, the Sinkhorn algorithm as follows:

Let C be the cost matrix, and a and b be the marginal distributions for the two groups. Define:


(1) $$K=\exp\left(-\lambda_{\mathrm{reg}}\cdot C\right)$$

The iterative updates for vectors *u* and *v* proceed as:


(2) u^(k+1)=a/(K v^k)

(3) v^(k+1)= b/(K^T u^(k+1))

where division is performed element-wise, and *k* denotes the iteration index. The final transport plan Π is then:


(4) Π=diag(u)Kdiag(v)

This plan is subsequently normalized across each row to ensure valid probability distributions for the mapped data. By multiplying the normalized transport plan with the raw expression data for Group 1, we obtain the “transported” expression profiles that align with the cells in Group 0. The resulting profiles facilitate a direct comparison of estimated gene expression between the two groups in a manner that accounts for distribution differences.

Finally, the transported expression profiles are subtracted from the original Group 0 expression values to yield per-gene differences. An average of these differences is then computed across cells to capture the overall directional shift in each gene’s expression, followed by a standard error calculation to assess variability. Wald statistics are performed by comparing the mean difference with zero (i.e. assuming no difference), providing a manner that quantifies the significance of each gene’s differential expression. Corresponding *P*-values are adjusted by Bonferroni correction, ensuring robust control of type I error.

### 2.2 Simulated datasets preparation

To simulate the data, we defined the following parameters: 10 samples (four assigned to Group 0, six to Group 1), each containing exactly 500 cells, with 2500 genes per dataset. A target sparsity level of 0.95 was set to mimic real scRNA-seq and 0.99 to scATAC-seq data characteristics.

We next generated base mean expression levels for all genes using a Gamma distribution. The average expression varies in different simulated datasets, from 10 read counts per gene to 1000. To model overdispersion, we sampled dispersion parameter *θ* from a Gamma distribution (Baruzzo *et al.* 2020, Svensson 2020) (shape = 2.0, scale = 1.0), ensuring sufficient variability to capture single-cell expression heterogeneity. Expression counts were simulated using negative binomial distributions for scRNA-seq data and Poisson distributions for scATAC-seq data (Wang *et al.* 2019). To take sample heterogeneity into account, we randomly given a small scaling factor from 0.9 to 1.1 on gene expression level per sample.

DEGs were specified by randomly selecting 200 genes, where 100 designated as upregulated and the remaining as downregulated. To introduce biologically relevant variation and mimic multi-modal (multi-peak) expression distributions, we applied a stepwise scaling strategy to cells in Group 0. Specifically, in the first step, 50% of the cells were randomly selected and scaled using predefined factors uniformly sampled from [1.1, 8.0] for upregulated genes and [0.1, 0.9] for downregulated genes in Group 1. In subsequent steps, 50% of the cells from the previous step were again selected and subjected to different scaling factors drawn from the same ranges. This recursive process continued for two times, with each successive subset receiving new multiplicative scaling values. As a result, the expression distributions of DEGs became increasingly multi-modal, effectively capturing the heterogeneity observed in real single-cell data.

Additionally, we implemented an expression-dependent dropout mechanism with a rate of 0.95. Dropout probabilities were calculated:


P(dropout)=(1-log(count+1)/max(log(count+1)))×0.95

This ensures lower-expressed genes had higher dropout probabilities. Counts were set to zero when uniform random values fell below these probabilities, accurately mimicking technical dropout in single-cell technologies. All simulation parameters and random seeds are provided in Supplementary Table 2, available as supplementary data at *Bioinformatics* online, and our code repository for full reproducibility.

### 2.3 Application of DEG prediction methods on artificial datasets

We applied OTMODE along with other 15 methods to perform DEA. These methods are carefully assigned into six categories, which are statistical testing category (e.g. *T*-test, Wilcoxon rank-sum test, overestimated variance *T*-test, and Memento), regression-based category (i.e. NB regression, logistic regression, Poisson regression, Monocle3 and MAST-glm), Bayesian-involved category (i.e. MAST-bayesglm and DESeq2), model comparison category (i.e. LRT), OT-based category (i.e. OTMODE and EMDomics (Nabavi *et al.* 2016)), and deep learning-based category (i.e. scVI (Gayoso *et al.* 2022)). Methods from statistical testing category and regression-based manner category except for Monocle3 and MAST were invoked by Scanpy ‘rank_genes_groups’ function and SciPy v1.14.1 Python toolkit (Virtanen *et al.* 2020). Memento leverages method-of-moments framework for scRNA-seq differential expression detection by matching moments across conditions, enabling fast testing. Additionally, a brief description of Monocle3, DESeq2, MAST, and EMDomics can be found in Wang *et al.* (2019). For the DEA algorithm of scVI we refer to Gayoso *et al.* (2022). For those R-based toolkits, we utilized zellkonverter v1.16.0 ‘readH5AD’ function (Zappia *et al.* 2021) to read datasets in R. Summary of the method description and belonged package is given in Supplementary Table 3, available as supplementary data at *Bioinformatics* online.

To examine the performance of OTMODE in differential features detection, we utilized multiple metrics from various angles to evaluate, which includes:

1. Distribution of log10-transformed *P*-values and volcano plots *P*-values and effect sizes. Ideally, the *P*-value distribution should have the following shape: (1) A flat/near-uniform region for *P*-values close to 1 (representing non-DEGs); (2) An upward spike near 0 (representing DEGs).
2. Correlation of log10-transformed *P*-values between different methods and Venn plot (Yang *et al.* 2024) of detected DEGs to evaluate the consistency of different detection outcomes.
3. Calculation of precision, recall, *F*1 score, accuracy, and AUC score to evaluate model performance in (1) the number of the predicted positive cases are actually positive; (2) the number of the actual positive cases were correctly predicted; (3) the proportion of correct predictions out of the total predictions; and (4) the ability to distinguish between positive and negative classes across various classification thresholds.
4. Comparison of analytical duration between OTMODE and other OT-based methods under identical hardware configuration to evaluate their computational efficiencies.

### 2.4 Real scRNA-seq data processing

Since the data has been pre-processed, we focused on the classical monocytes and performed DEA using Scanpy and OTMODE (*λ* = 0.1). For OTMODE, we utilized top 15 PCs as an input for Gaussian kernel density estimation to model the marginal distribution of two groups. *P*-values are further corrected by Bonferroni correction. Genes are considered differentially expressed if the adjusted *P*-value is below .05. The results were further visualized through volcano plots, and their overlapping genes were demonstrated by Venn plot. DEGs were recruited to identify significant pathways using Enrichr (Kuleshov *et al.* 2016) with 2021 KEGG Human repository (Kanehisa *et al.* 2021). Protein networking was constructed by STRING database (Szklarczyk *et al.* 2023) using the threshold of 0.4.

### 2.5 Real scATAC-seq data processing

In addition to scRNA-seq analysis, scATAC-seq data processing were also conducted. We followed the guidance of Zhang *et al.* (2024), encompassing steps such as pre-processing (e.g. low-quality cell and peak filtering, doublet removal, outlier detection, and feature selection), dimensionality reduction (e.g. spectral embedding and UMAP), Leiden clustering, cell-type annotation, and peaking calling. This pre-processing pipeline is primarily built upon SnapATAC2 and integrated with Scrublet (Wolock *et al.* 2019) for doublet detection. Specifically, cells were excluded if their transcription start site enrichment score was less than 10 or if their peak count was less than 5000 or greater than 100 000. Top 250 000 highly variable features were selected for subsequent analysis. Resolution of Leiden clustering was set 0.5. We later selected naïve B cells and memory B cells for DAR identification using both SnapATAC2 and OTMODE (*λ *= 0.1), followed by Bonferroni correction. For OTMODE, we utilized top 15 PCs as an input for Gaussian kernel density estimation to model the marginal distribution of two groups. Accessible regions were considered differentially abundant the adjusted *P*-value is below .05. TF enrichment analysis was performed using the results from both DAR detection methods by SnapATAC2 ‘snap.tl.motif_enrichment’ function under default settings.

## 3 Results

### 3.1 Overview of the OTMODE and the impact of OT parameter on its performance

**Figure 1.**
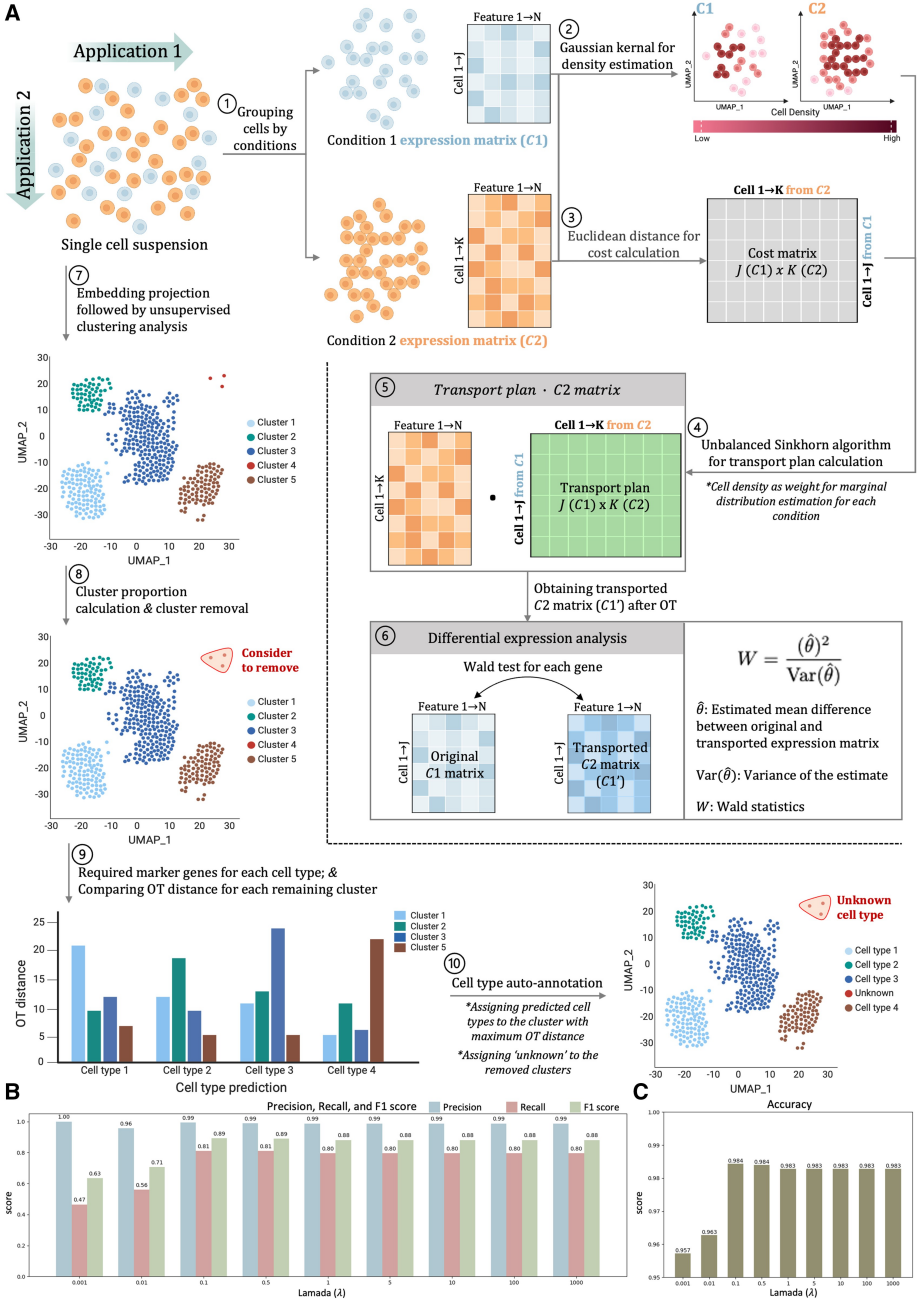
Architecture of the OTMODE. (A) The illustrative schematic showcases two applications of the OTMODE. Application 1 serves as an approach designed for detecting differential features between conditions where unbalanced Sinkhorn algorithm was applied to calculate transport plan. The significant features are identified by comparing the transported condition 2 matrix (C1′) and original condition 1 matrix (C1) using Wald test, where follows a standard normal distribution. Application 2 facilitates cell-type annotation by quantifying the effect of multiple genes specifically expressed in a certain cell type. This quantification can be represented by OTD. Clusters are assigned cell types by the maximum distance among predicted cell types. (B) Barplot for comparing precision, recall, and *F*1 score under different regularization strengths. (C) Barplot for accuracy comparison under different regularization strengths (*λ* in *x*-axis).

OTMODE comprises two main applications (Fig. 1A): (1) detecting differential features between conditions in single-cell multi-omics data, and (2) enhancing cell-type annotation. For differential analysis, input data are divided into two condition-specific matrices, C1 and C2, representing distinct biological states. Gaussian kernel density estimation is applied to each matrix to emphasize densely clustered cells, which are assumed to share similar biological properties. A cost matrix is then computed using Euclidean distances between cells in C1 and C2. These cell densities and the cost matrix are fed into the unbalanced Sinkhorn algorithm, an entropy-regularized OT method, to estimate a transport plan mapping cells between conditions. Applying this plan to C2 yields a “transported” matrix C1′, aligned with C1 for direct comparison. Finally, Wald test is performed on each feature between C1 and C1′ to identify significant differences, while accounting for sample imbalance.

In the annotation step, cells are first clustered using unsupervised methods, with low-proportion clusters filtered out. For each remaining cluster, the unbalanced Sinkhorn algorithm computes the annotation score (OT distance; OTD) to every other cluster, using a flexible set of marker genes, including both positive and negative markers. We hypothesize that authentic cell types show higher OTDs when compared to unrelated clusters, reflecting distinct distributional profiles. Statistical significance is assessed via permutation test. A cluster is assigned to a specific cell type only if it (1) shows the highest OTD among all clusters for that cell type, and (2) achieves a significant *P*-value of permutation test. Clusters with low cell proportions or ambiguous results are labeled as “unknown” for further refinement. We also provided two pseudocodes for better illustration (Supplementary Fig. S1, available as supplementary data at *Bioinformatics* online).

We evaluated the effect of regularization strength (*λ*) on model performance using both simulated scRNA-seq and scATAC-seq data (Section 2). *λ* was varied from 0.001 to 1000, and performance was assessed using precision, recall, accuracy, and *F*1 score. Precision remained stable across *λ* values, indicating effective control of false positives. While higher *λ* values improved DEG detection accuracy, optimal performance was consistently achieved with *λ* between 0.5 and 5 for both modalities (Fig. 1B and C; Supplementary Fig. S3A and B, available as supplementary data at *Bioinformatics* online), providing a practical guideline for selecting *λ*.

### 3.2 OTMODE is more sensitive than other DEG detection methods, meanwhile showing good false-positive control

**Figure 2.**
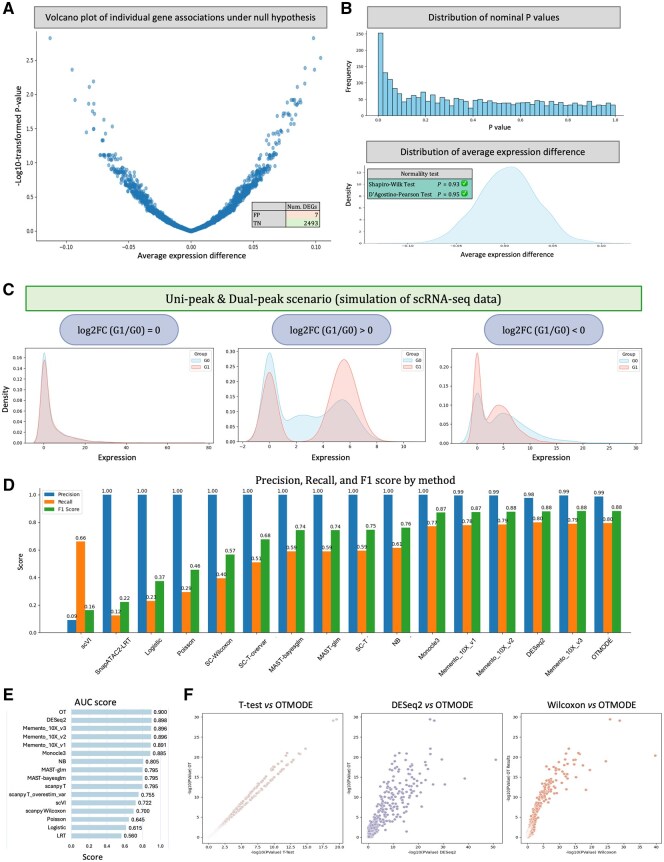
OTMODE outperforms other DEG detection methods, meanwhile showing good false-positive control. (A) Volcano plot of DEGs in simulated scRNA data where no DEG was assigned. (B) Density plot of average expression difference for each gene and distribution of nominal *P*-values of genes. (C) Simulated scRNA-seq expression distributions for different log2 fold change (log2FC) scenarios. Each panel compares the expression density of two groups (G1 and G0) to illustrate the presence or absence of differential expression. (D) Comparison among 16 DEA manners using precision, recall, and *F*1 score in simulated scRNA-seq data. The 16 manners are order by the *F*1 score. NB: negative binominal. (E) Ranked AUC score across 16 methods. The *x*-axis is scaled between 0.5 to 1.0. (F) Correlation of log10-transformed *P*-values between OTMODE (*y*-axis) and other three methods. The order from left to right is *T*-test, DESeq2, and Wilcoxon sum-rank test.

To evaluate the robustness of OTMODE against sample or cellular heterogeneity, we applied the method to an independently simulated dataset containing no DEGs, with an overall sparsity of 44.7%. Remarkably, only seven out of 2500 genes were falsely identified as DEGs following Bonferroni correction (Fig. 2A), indicating a low false-positive rate. Furthermore, the distribution of *P*-values was approximately uniform (Fig. 2B), consistent with expectations under the null hypothesis and indicative of appropriate type I error control. The average gene expression values followed a standard normal distribution (Fig. 2B), as confirmed by two independent normality tests (Shapiro–Wilk *P* = .93; D’Agostino–Pearson *P* = .95). These results collectively support the statistical robustness of OTMODE under null conditions.

We next assessed the performance of OTMODE using an the primary simulated scRNA-seq dataset, with an overall sparsity of 44.5%. To benchmark its effectiveness, we compared OTMODE against 15 widely used differential expression analysis (DEA) methods. To closely approximate the complex expression patterns observed in real scRNA-seq data, we simulated three distinct expression scenarios (Fig. 2C): (1) a uni-peak or dual-peak distribution representing no differential expression (logFC = 0), (2) a dual-peak distribution simulating upregulated genes (logFC > 0), and (3) a dual-peak distribution simulating downregulated gene (logFC < 0). These scenarios were designed to rigorously challenge the sensitivity and specificity of each method under realistic expression dynamics.

OTMODE successfully identified 161 out of 200 simulated DEGs, even when fold changes were modest (<1.5), highlighting its sensitivity to subtle transcriptional differences. As shown in Fig. 2D, OTMODE achieved the highest *F*1 score (0.88) among all the methods, reflecting an optimal balance between high precision (0.99) and robust recall (0.80). Furthermore, it attained the highest area under the receiver operating characteristic (ROC) curve (AUC = 0.94; Fig. 2E), underscoring its superior classification accuracy across a range of expression levels. Collectively, these results demonstrate that OTMODE is not only highly sensitive and specific but also robust across diverse expression scenarios. Notably, OTMODE outperformed several widely used methods, including MAST, and exhibited comparable or superior performance relative to well-established tools such as DESeq2 and Monocle3.

To further validate its performance, we compared OTMODE with three widely used methods, *t*-test, Wilcoxon rank-sum test, and DESeq2, by examining the correlations of log_10_-transformed *P*-values. These methods represent diverse statistical paradigms commonly used in the field. As shown in Fig. 2F, OTMODE exhibited strong positive correlations with all three approaches, indicating consistent DEG detection across methodologies. Among them, OTMODE appeared to demonstrate higher overall sensitivity, further supporting its effectiveness in detecting subtle transcriptional changes.

### 3.3 OTMODE reveals novel biological insights in real-world case

**Figure 3.**
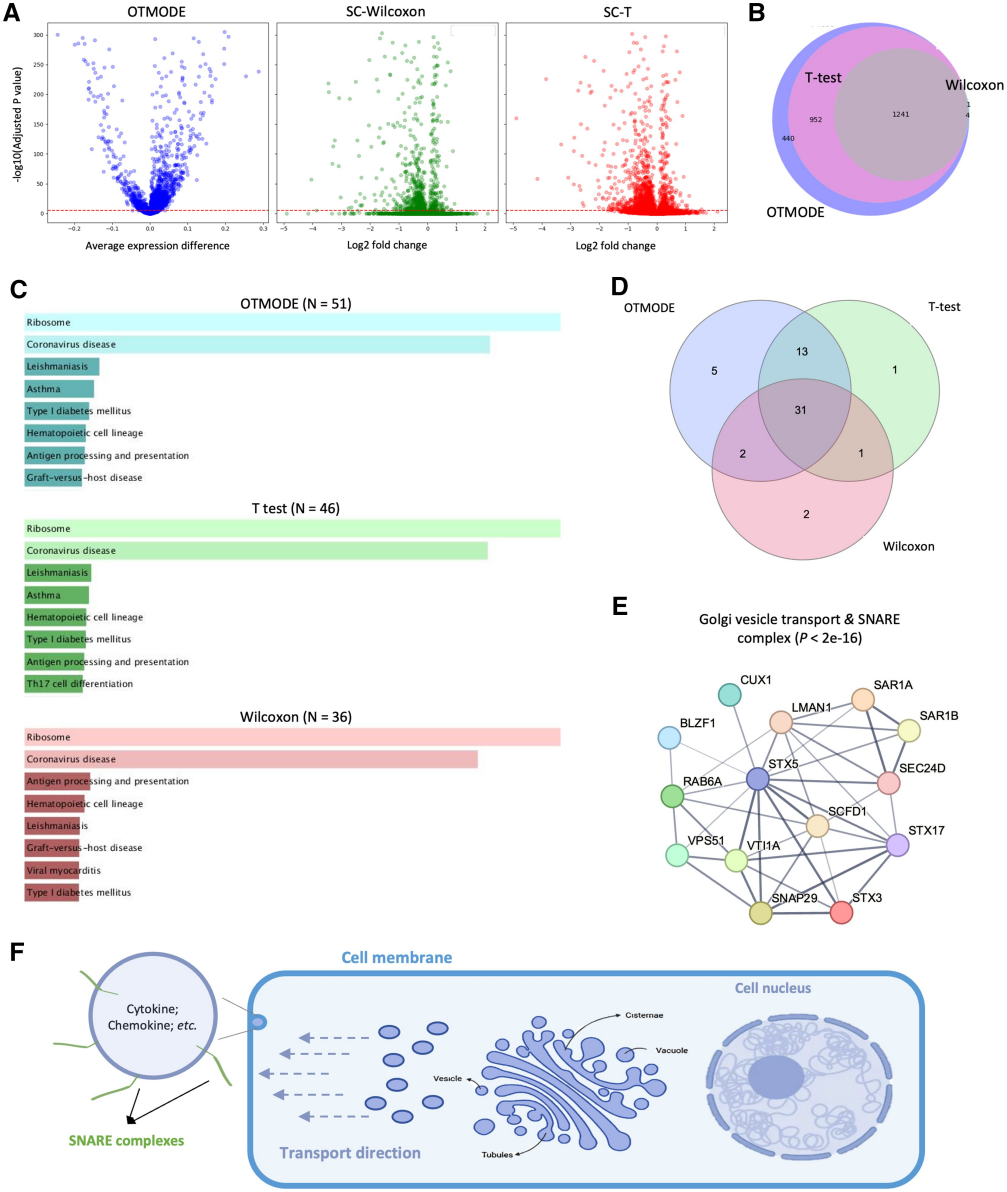
OTMODE discovered biological meaningful information using real scRNA-seq data. (A) Volcano plots for all genes in classic monocytes between healthy and SLE samples in OTMODE, and Wilcoxon sum-rank test, and *T*-test method from left to right. Red horizontal dotted line indicates the *P*-value threshold (*α* = 0.05/13243). (B) Venn plot to show the relation of DEGs identified by the three methods. (C) GO pathway analysis using the upregulated genes in three methods. For clarity, only top 10 pathways are given. GO: gene ontology. (D) Venn plot of all significant pathways identified from three methods. (E) Protein networking for the genes enriched in ‘Golgi vesicle transport and SNARE complex’ pathway. (F) An illustration of the function of SNARE complexes at sub-cellular level.

We next applied OTMODE, *t*-test, and Wilcoxon rank-sum test to a publicly available scRNA-seq dataset generated from peripheral blood mononuclear cells (PBMCs; Supplementary Fig. S2, available as supplementary data at *Bioinformatics* online; Wang *et al.* 2024) We performed DEA on classical monocytes from both systemic lupus erythematosus (SLE) patients and controls. After Bonferroni correction, OTMODE identified 2633 significant DEGs, more than those detected by *t*-test (*N* = 2193) and Wilcoxon test (*N* = 1241) (Fig. 3A), with substantial overlap among the methods (Fig. 3B; Supplementary Data 3–5, available as supplementary data at *Bioinformatics* online). We also attempted to apply DESeq2 via Python but were unsuccessful due to environmental dependency conflicts.

To evaluate biological relevance of the findings, we conducted pathway enrichment analysis on upregulated genes in SLE (Supplementary Data 6, available as supplementary data at *Bioinformatics* online). The top 10 enriched pathways identified were highly consistent among the three methods (Fig. 3C), with notable overlap in significant terms (Fig. 3D, Jaccard index: OTMODE versus *t*-test = 0.83; OTMODE versus Wilcoxon = 0.62). Notably, OTMODE uniquely identified the SNARE-related pathway as significantly upregulated in SLE, involving 14 genes (Fig. 3E; Supplementary Data 7, available as supplementary data at *Bioinformatics* online). This pathway, essential for vesicle transport and exocytosis (Kasai *et al.* 2012; Fig. 3F) was not detected by the other methods, likely due to their lower sensitivity.

### 3.4 OTMODE shows excellent performance on handling data with large heterogeneity and sparsity

**Figure 4.**
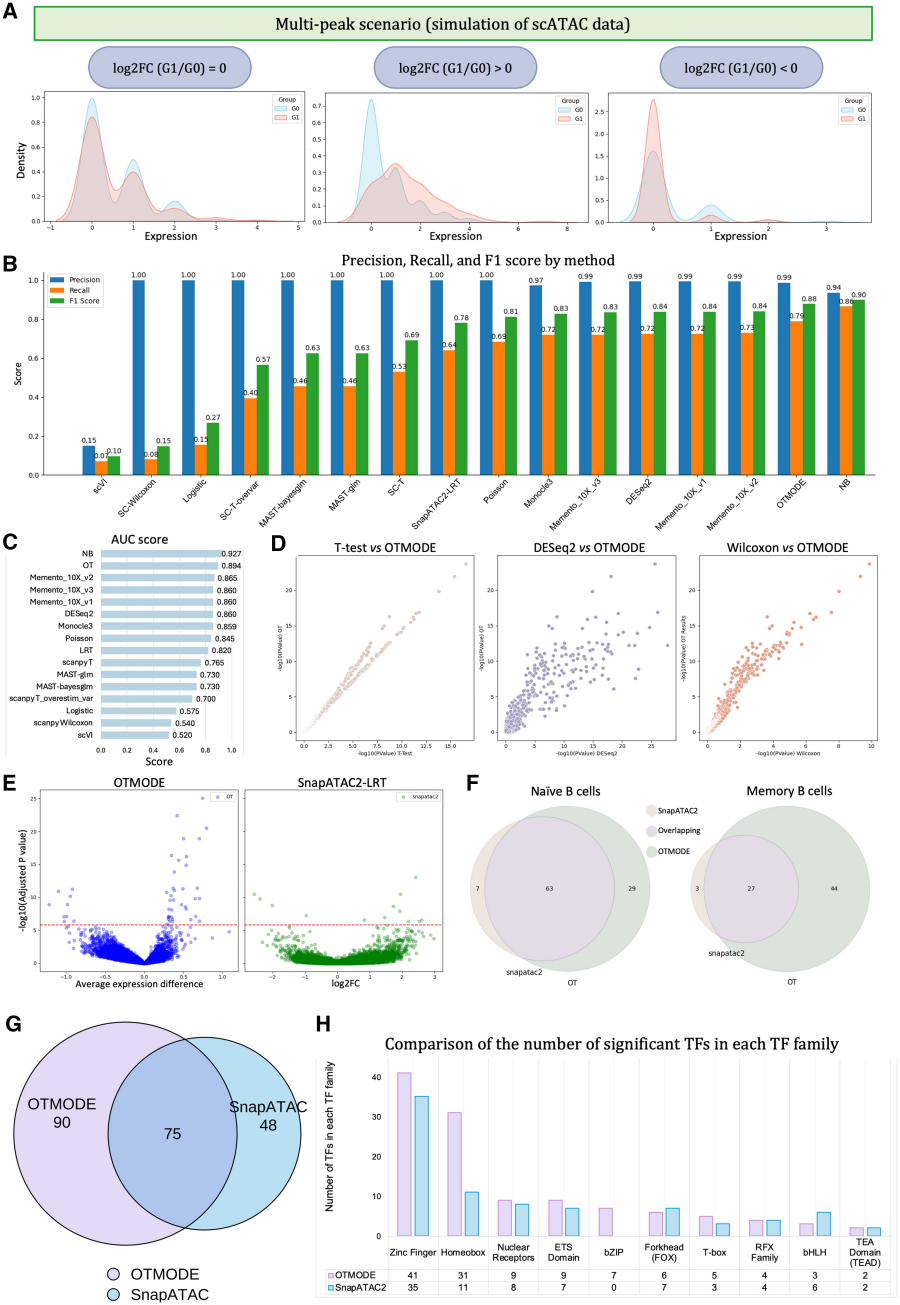
OTMODE shows excellent generalizability on differential analysis in multi-omics data. (A) Simulated scATAC-seq expression distributions for different log2FC scenarios. (B) Comparison among 16 DAA manners using precision, recall, and *F*1 score in simulated scATAC-seq data. The 16 manners are order by the *F*1 score. NB: negative binominal. (C) AUC score for each method. (D) Correlation of log10-transformed *P*-values between OTMODE (*y*-axis) and other three methods. The order from left to right is *T*-test, DESeq2, and Wilcoxon sum-rank test. (E) Volcano plots for peaks between memory and naïve B cells in OTMODE and SnapATAC2 method from left to right. Red horizontal dotted line indicates the threshold of adjusted *P*-value (*α* = 0.05/32361). (F) Venn plot to show the relation of DARs identified by the two methods. (G) Venn plot to show the relation of identified TFs enriched in memory B cells by the two methods. (H) Bar plots presentation of the number of significant TFs in top 10 TF families. bZIP: basic leucine zipper domain; RFX: regulatory factor X; ETS-domain: erythroblast transformation specific domain; bHLH: basic helix–loop–helix.

One of OTMODE’s key advantages is the adaptability to diverse data types. To assess its performance on scATAC-seq data, we employed a primary simulated scATAC-seq dataset, characterized by high sparsity (82.2%) and multi-peak distributions. We evaluated performance across three differential scenarios defined by multimodal feature distributions (Fig. 4A). Among 16 tools tested, OTMODE achieved the second-highest *F*1 score (0.88), with precision of 0.99 and recall of 0.79, outperforming state-of-the-art tools such as DESeq2 and Monocle3 (Fig. 4B). While its *F*1 score was slightly lower than that of the negative binomial regression (NB) method, OTMODE exhibited higher precision (0.99 versus 0.94), indicating a stronger ability to reduce false positives. It also attained the second-highest AUC (0.90; Fig. 4C), highlighting its robust classification performance across a range of chromatin accessibility levels. Notably, the likelihood ratio test (LRT), used by default in SnapATAC2, demonstrated lower sensitivity in this context. Finally, correlation analysis of log-transformed *P*-values (Fig. 4D) showed strong consistence between OTMODE and three alternative methods, further supporting its reliability for chromatin accessibility data at single-cell level.

We next tested OTMODE to real scATAC-seq data from healthy human PBMCs to identify differentially accessible regions (DARs) between naïve and memory B cells (Supplementary Fig. S3, available as supplementary data at *Bioinformatics* online; Zhang *et al.* 2024). OTMODE detected more significant DARs than SnapATAC2 (163 versus 100), covering 90% of SnapATAC2's results under Bonferroni correction (Fig. 4E and F; Supplementary Data 8 and 9, available as supplementary data at *Bioinformatics* online). We hence compared the transcription factors (TFs) enriched in memory B cells by mapping known TF binding motifs into the accessible chromatin regions (165 versus 123; Supplementary Data 10 and 11, available as supplementary data at *Bioinformatics* online), capturing 60.9% of those reported by SnapATAC2 (Fig. 4G). Grouping TFs by gene family revealed largely consistence on the top 10 families across both methods, but OTMODE detected more significant TFs within each TF family, underscoring its advantage in capturing regulatory signals (Fig. 4H; Supplementary Data 12, available as supplementary data at *Bioinformatics* online). Uniquely, OTMODE identified a TF family, bZIP, where six TFs were enriched in memory B cells. In contrast, SnapATAC2 did not identify this TF family.

### 3.5 OTMODE showed much better performance and efficiency than EMDomics, another OT-based tool

**Figure 5.**
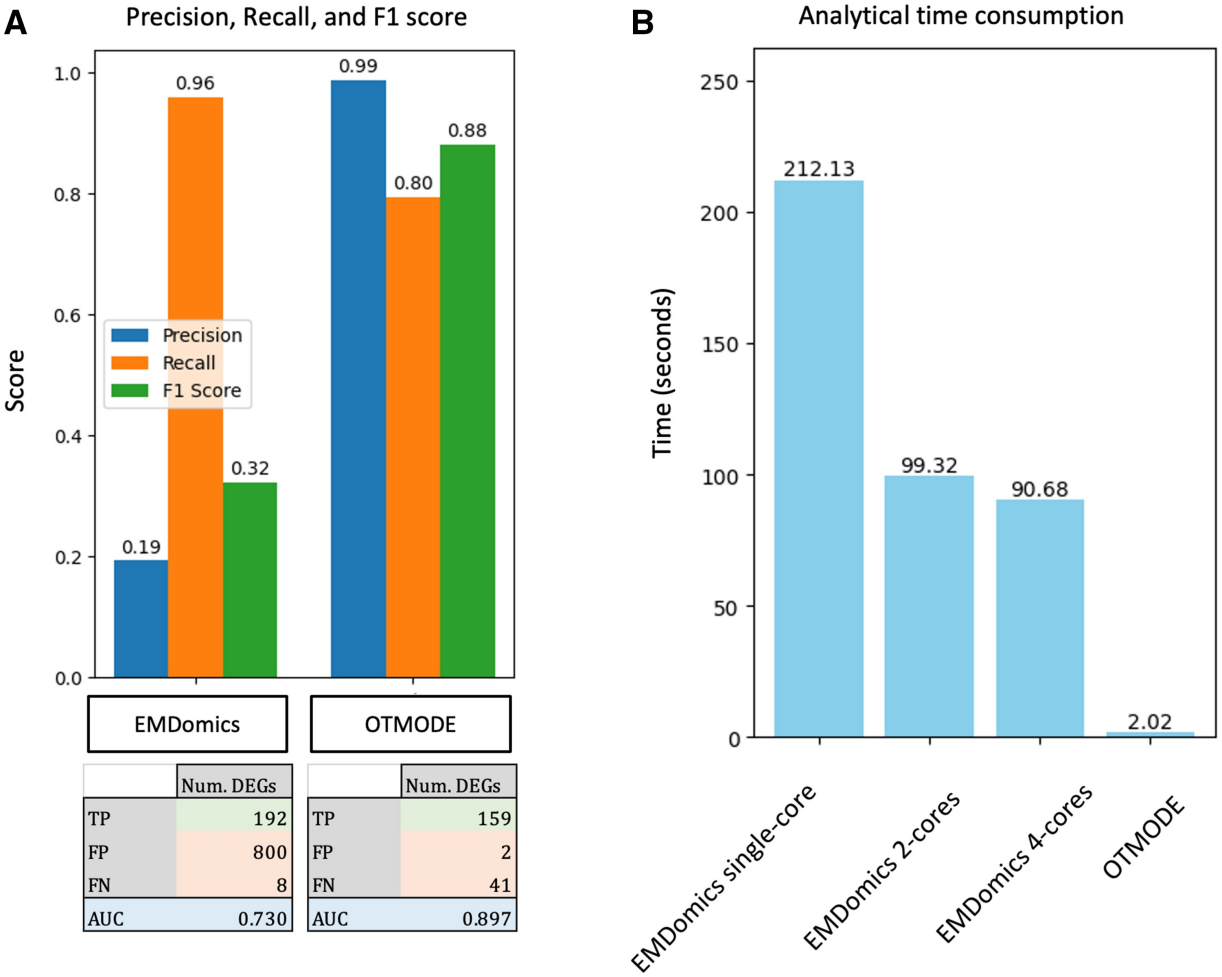
OTMODE surpasses other OT-based methods. (A) Comparison between OTMODE and EMDomics using precision, recall, *F*1 score, and AUC score in simulated scRNA-seq data. (B) Analytical duration of DEA (Ncells = 5000) under various computational resource allocations.

We benchmarked OTMODE against another OT-based method, EMDomics, using the primary simulated scRNA-seq dataset. While EMDomics identified slightly more DEGs (192 versus 161 out of 200), OTMODE achieved markedly higher precision and *F*1 score by substantially reducing false positives (2 versus 800; Fig. 5A). OTMODE also demonstrated superior classification performance, with an AUC of 0.90 compared to 0.73 for EMDomics. In terms of computational efficiency, OTMODE completed the analysis in just 2.0 s using a single core on a Mac mini with an M2 chip. In contrast, EMDomics required 90.7 s, despite utilizing four-core parallelization on the same hardware (Fig. 5B).

### 3.6 OTMODE facilitates cell-type annotation by measuring OTDs across clusters

OTMODE has the advantage to improve annotation accuracy. We first applied OTMODE to a real scRNA-seq dataset comprising 3000 PBMCs. The data were preprocessed following the protocol outlined by Heumos *et al.* (2023) and Louvain clustering was performed at a resolution of 0.5, which provides a balanced level of detail when identifying distinct cell groups (Supplementary Fig. S4A and B, available as supplementary data at *Bioinformatics* online). To assess OTMODE’s performance, we manually annotated clusters using known marker genes from the original study (Supplementary Fig. S4C, available as supplementary data at *Bioinformatics* online). These marker genes were then input into OTMODE to compute the OTD, followed by a permutation test (*N* = 100). The results were visualized as a bar plot, where each bar represents a cluster (i.e. predicted cell type), and bar height corresponds to the OTD score (Supplementary Fig. S4D, available as supplementary data at *Bioinformatics* online). Comparing OTMODE’s annotations with manual labels using established cell markers confirmed its high accuracy in cell-type identification (Supplementary Fig. S4E, available as supplementary data at *Bioinformatics* online). Notably, some marker genes, such as those for NK cells and CD8^+^ T cells, expressed across multiple clusters. OTMODE effectively resolved this by integrating the quantitative contributions of multiple markers into a unified metric, enabling consistent and improved interpretation.

**Figure 6.**
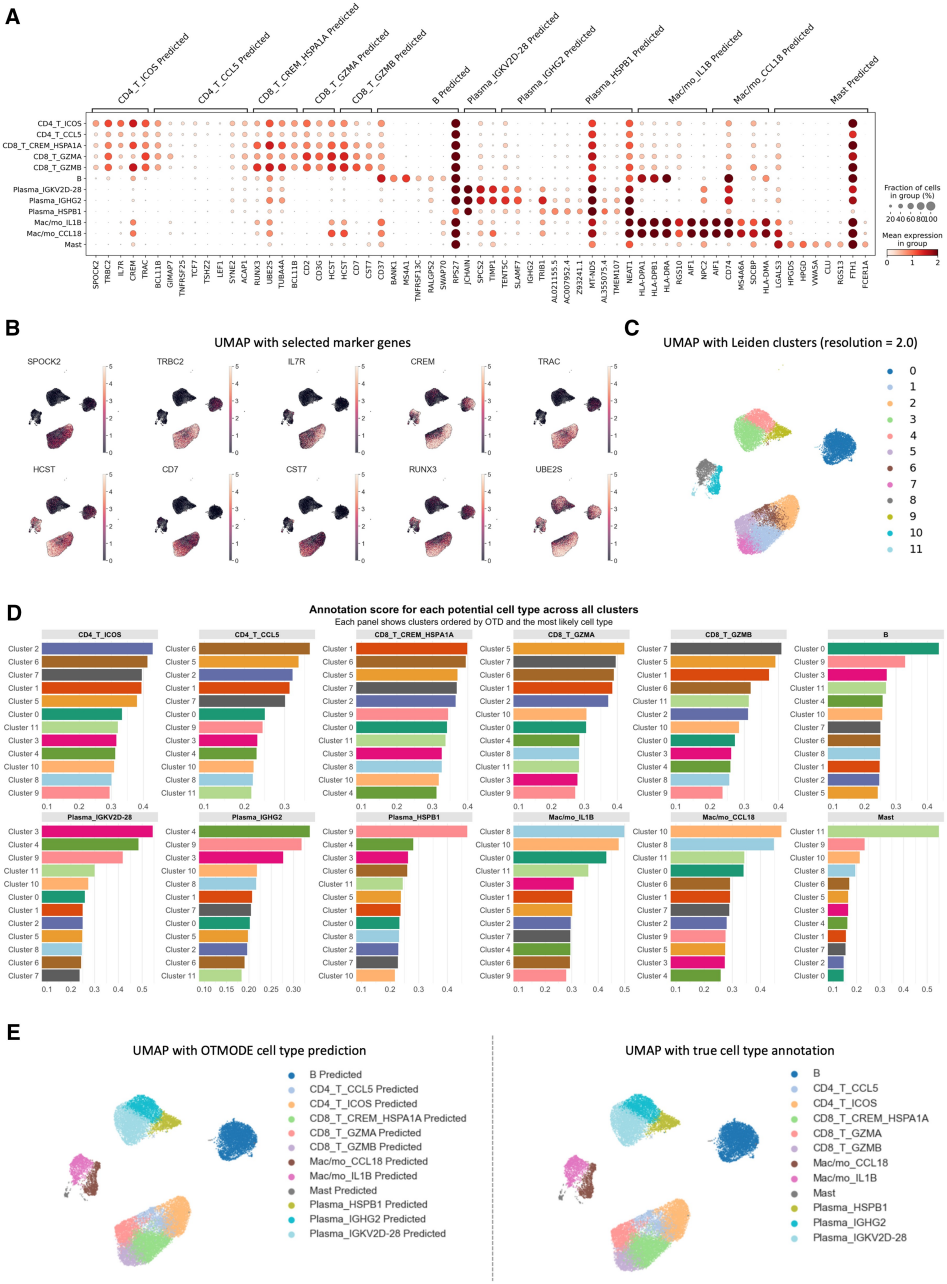
OTMODE distinguishes cell subgroups by integrating multiple marker genes. (A) Dot plot showing the expression of marker genes from the original study across both predicted and annotated cell types. (B) UMAP visualization of Leiden clusters, with clusters distinguished by color. (C) UMAP plots displaying the expression patterns of selected marker genes across all cells. (D) OTMODE annotation scores for each cluster across potential cell types, based on OTD. Each panel represents a predicted cell type, with bars indicating clusters ranked by their OTD score. Higher scores indicate stronger alignment with the corresponding cell type. The *x*-axis represents the OTD score. € Comparison of UMAPs colored by cell types predicted by OTMODE (left) and ground truth annotations (right).

Next, we evaluated the method’s performance on a publicly available scRNA-seq dataset (Xiang *et al.* 2023) comprising 72 853 salivary gland cells from 11 primary Sjögren’s syndrome (pSS) patients and five controls, annotated at high resolution. We extracted 24 369 immune cells based on the original annotations. A dot plot of known marker genes revealed that several, such as *HCST* and *UBE2S*, were broadly expressed and lacked cell-type specificity (Fig. 6A). To explore this further, we visualized 10 marker genes on a UMAP and compared their expression with Leiden clustering (resolution = 2.0; Fig. 6B and C). The widespread expression of these markers complicated precise cell-type identification.

We then applied OTMODE using these markers to assess whether the cluster assignments were arbitrary. OTMODE ranks clusters by OTD, identifying the most representative cluster for each cell type (Fig. 6D). Notably, subgroups of CD8^+^ T cells exhibited similar OTD scores and spatial proximity on the UMAP, suggesting overlapping transcriptional profiles, while MAST cells were more distinct, reflecting their functional specificity. OTMODE annotations closely aligned with the original labels (Fig. 6E). In contrast, CellTypist performed less effectively: (1) several clusters were assigned multiple cell types (Supplementary Fig. S5A, available as supplementary data at *Bioinformatics* online); and (2) cell types appeared more dispersed on the UMAP (Supplementary Figs. S5A and B, available as supplementary data at *Bioinformatics* online).

## 4 Discussion

OTMODE provides a universal framework for efficiently identifying differential features in single-cell multi-omics data by optimizing the minimal cost between two probability distributions. This approach enables the detection of non-uniform feature differences across conditions at single-cell resolution. In benchmarking analyses, OTMODE consistently outperformed existing methods and demonstrated robust performance across simulated scRNA-seq and scATAC-seq datasets. Notably, it maintained strong performance under high sparsity, a common challenge in single-cell analyses, and achieved a low false-positive rate (0.28%) in simulations without DEGs, highlighting its reliability in heterogeneous data architectures.

In real-world applications, OTMODE demonstrated superior sensitivity, identifying a greater number of differential features between the groups compared to existing methods. Importantly, it not only detected additional novel signals but also successfully recovered the majority of key features identified by widely used tools such as Scanpy and SnapATAC2, highlighting both its robustness and the potential for novel findings. The comprehensive feature detection capability of OTMODE showcases its versatility and extensibility across diverse omics applications, ranging from cell cycle prediction tools like MomicPred (Shi *et al.* 2025) and scHiCyclePred (Wu *et al.* 2024) to specialized classification methods such as scHiClassifier (Zhou and Wu 2024) for identifying cell types from Hi-C data. Pathway analyses further revealed that the OTMODE-identified features were enriched in established pathways for disease pathogenesis and progression. For instance, in SLE scRNA-seq data, OTMODE highlighted the role of SNARE complex transport in monocytes, consistent with previous experimental findings (Kasai *et al.* 2012, Hirose *et al.* 2019, Ożańska *et al.* 2020). Similarly, in scATAC-seq data from the healthy human controls, OTMODE-detected TFs from the *HOX* and *FOX* gene families, both of which have established roles in B cell development and maturation (Bertrand *et al.* 2003, Hu *et al.* 2006). Notably, OTMODE uniquely highlighted members of the bZIP TF family, which have been implicated in the regulation of long-term memory formation in human (Shaulian and Karin 2002, Nerlov 2007, Walter and Ron 2011, Luo *et al.* 2012). Recent studies further suggest that the expression levels of bZIP TFs play a pivotal role in shaping memory B cell fate decisions (Moroney *et al.* 2020, Shao *et al.* 2024). Therefore, our findings underscored that the potential of OTMODE not only to align with established methods but also to reveal biologically meaningful signals that may otherwise be overlooked.

OTMODE introduces several enhancements to improve differential feature detection and facilitate broader applicability in single-cell analysis. First, we propose a novel metric, OTD, which leverages OTMODE’s high sensitivity to improve cluster annotation by delineating subpopulations of cells. This metric provides a robust framework for evaluating annotation accuracy and supports more nuanced biological interpretation, complementing clustering approaches like JLONMFSC (Lan *et al.* 2024b) that employ joint learning of non-negative matrix factorization and subspace clustering. Second, we designed a versatile and modular framework that integrates seamlessly with the Scanpy and the scverse ecosystem (Virshup *et al.* 2023). Third, unlike many of the current tools, including DESeq2, Monocle3, and MAST, OTMODE can directly access the expression matrix from the AnnData object (Virshup et al. 2021) and store results in the AnnData.uns slot, providing an easy-to-use operation and streamlining the analysis workflow. Finally, significance is assessed using p-values from Wald test, simplifying result interpretation.

Our comparative analysis with EMDomics revealed key algorithmic differences explaining OTMODE’s superior performance. First, OTMODE employs entropy-regularized OT via the Sinkhorn algorithm, providing numerical stability and preventing overfitting in sparse single-cell data, whereas EMDomics uses unregularized Earth Mover’s Distance. Also, OTMODE’s Wald statistics enable rapid significance testing compared to EMDomics’ computationally intensive permutation approach. These designs explain why OTMODE achieved an extraordinary reduction in false positives while maintaining high computational efficiency, demonstrating its robustness for single-cell multi-omics analysis.

To support large-scale datasets, OTMODE incorporates GPU acceleration through its implementation in the POT framework (Flamary *et al.* 2021), which employs the Cupy backend (Nishino and Hido Crissman Loomis 2017) to ensure compatibility with GPU-based computations. By default, OTMODE automatically selects the most suitable backend, while also allowing users to manually configure it to optimize GPU memory usage. This flexibility enhances OTMODE’s adaptability across diverse computational settings, addressing the scalability challenges faced by transformer-based approaches (Lan *et al.* 2024a) and large language models in biomedical applications (Lan *et al.* 2025), which often require substantial computational resources for training and inference.

Despite its strengths, OTMODE has several limitations. First, simulations, while useful for benchmarking, may over-simplify biological complexity. To address this, we tested OTMODE across diverse simulated datasets, where it consistently performed well. In real-world applications, such as scATAC-seq analysis of PBMCs, it identified more biologically relevant features than existing methods, supporting conclusions from simulation-based results. Second, OTMODE-detected features demonstrated higher statistical significance, indicating strong sensitivity to subtle signals. Furthermore, extensive benchmarking also confirmed its ability to control false discovery rates. Third, OTMODE currently lacks covariate adjustment; we recommend pre-correcting batch effects using ComBat (Johnson *et al.* 2007), which has demonstrated excellent performance in analysing both scRNA-seq and scATAC-seq datasets in recent benchmarking studies (Luecken *et al.* 2022, Antonsson and Melsted 2025). Finally, while deep generative models like scCross (Yang *et al.* 2024) enable cross-modal generation, OTMODE currently focuses on differential analysis within each modality so that future extensions could leverage OT for cross-modal integrations.

In conclusion, OTMODE represents a significant advance in single-cell multi-omics analysis by leveraging OT theory to address fundamental challenges in differential feature detection. By maintaining single-cell resolution while capturing complex expression patterns, OTMODE enables the discovery of subtle yet biologically meaningful signals that may be overlooked by existing methods. As single-cell technologies continue to evolve, OTMODE’s flexible framework and seamless integration with the scverse ecosystem position it as a valuable tool for advancing our understanding of cellular heterogeneity in health and disease, ultimately contributing to precision medicine approaches at single-cell resolution.

## Supplementary Material

btaf650_Supplementary_Data

## Data Availability

Eight scRNA data and scATAC-seq datasets for benchmarking purpose are simulated in Python, where the code for data generation is provided. Real raw human SLE scRNA-seq data (Nehar-Belaid *et al.* 2020) is from GEO GSE135779. Real scATAC-seq data is from https://cf.10xgenomics.com/samples/cell-atac/1.2.0/atac_pbmc_5k_nextgem/atac_pbmc_5k_nextgem_web_summary.html (healthy human 5k PBMCs, 10x Genomics). Reference genome for peak identification is from https://www.ncbi.nlm.nih.gov/assembly/88331 (Human Genome GRCh38). Database for motif enrichment is from Weirauch *et al.* (2014), and can be accessed from https://kzhang.org/SnapATAC2/api/_autosummary/snapatac2.datasets.cis_bp.html (reformatted CIS-BP database by SnapATAC2 (Zhang *et al.* 2024)). Real pbmc scRNA-seq data for annotation purpose is from https://www.10xgenomics.com/datasets/3-k-pbm-cs-from-a-healthy-donor-1-standard-1-1-0 (Healthy human 3k PBMCs, 10X Genomics). Processed pSS scRNA-seq data for annotation is from https://zenodo.org/records/10884425. The cell-type marker data was obtained from the Azimuth database (Hao *et al.* 2024) (available at: https://azimuth.hubmapconsortium.org/references/#Human%20-%20PBMC) and CellTypist Encyclopedia Immune v2 (available at: https://www.celltypist.org/encyclopedia/Immune/v2).
