# Supplementary material for: OTMODE: an optimal transport theory-based framework for identifying differential features in single-cell multi-omics data
Source: Bioinformatics. 2025 Dec 3;42(1):btaf650. doi: 10.1093/bioinformatics/btaf650 (PMC12766913; doi:10.1093/bioinformatics/btaf650)
Supplement: btaf650_Supplementary_Data [file btaf650_supplementary_data.zip › OTMODE_Supp_materials.pdf]

OTMODE: An Optimal Transport Theory-Based Framework for Identifying Differential Features in Single-Cell Multi-Omics Data

Huidong Su[*1], Caicai Zhang[1], Frank Qingyun Wang[1], Chun Hing She[1], Xinxin Chen[1], Xiao Dang[1], Yao Lei[1], Ke Ni[2], Zewei Xiong[3], Danqing Yin[4,5], Xingtian Yang[1], Hong Feng[1], Philip H. Li[6], and **Wanling Yang**[†1]

[1]Department of Paediatrics and Adolescent Medicine, LKS Faculty of Medicine, The University of Hong Kong, Hong Kong SRA, China

[2]Joint Carnegie Mellon–University of Pittsburgh Program in Computational Biology, Pittsburgh, PA, USA

[3]Department of Psychiatry, Li Ka Shing Faculty of Medicine, The University of Hong Kong, Hong Kong SAR, China

[4]Laboratory of Data Discovery for Health Limited (D24H), Pak Shek Kok, Hong Kong SAR, China

[5]School of Biomedical Sciences, Li Ka Shing Faculty of Medicine, The University of Hong Kong, Pokfulam, Hong Kong SAR, China

[6]Division of Rheumatology & Clinical Immunology, Department of Medicine, Queen Mary Hospital, The University of Hong Kong, Hong Kong SAR, China

[*]First author

[†]Corresponding author: wlyang@hku.hk

Contents

1 Related work **3**
1.1 Single-cell DEG detection methods and their limitations 3
1.1.1 DEG detection at the single-Cell level 3
1.1.2 DEG detection at the pseudobulk level 4
1.2 Optimal transport in single-cell biology 4

2 Theoretical background **6**

3 OTMODE pseudocode **13**

4 Supplementary figures **14**

1 Related work

1.1 Single-cell DEG detection methods and their limitations

The historical, present, and forthcoming states of cells and tissues are hidden behind the abundance, diversity, and functionality of RNA molecules. RNA sequencing (RNA-seq), which enables the comprehensive quantification of messenger RNA (mRNA) species, has provided unparalleled insights into the molecular processes active within a biological sample. These processes are perturbed by diseases and experimental manipulations, which can be monitored by evaluating the changes of specific mRNAs (Handley *et al.,* 2015). Recent years, single cell technologies have flourished and revolutionized the field of mRNA quantification, making it possible to detect mRNA molecules at individual cells. The advancement of these technologies enables large-scale comparisons for each cell-type between non-perturbed and perturbed samples in complex tissues, offering the resolution needed to unveil cell-type specific responses to perturbations (Burkhardt *et al.,* 2021).

1.1.1 DEG detection at the single-Cell level

Despite the high resolution offered by single-cell RNA sequencing (scRNA-seq), current methods for differential gene expression (DEG) detection at the single-cell level face several critical challenges. A major difficulty lies in accurately modeling the inherently complex and heterogeneous nature of single-cell transcriptomic data. Specifically, many existing tools struggle with the sparse and multimodal distribution of gene expression values. Sparsity—caused by both technical dropout events and genuine biological variation—results in a high frequency of zero or near-zero counts, which can obscure true expression differences and inflate variance estimates. This makes it difficult to distinguish noise from biologically meaningful signals, particularly for genes expressed at low levels.

Furthermore, gene expression distributions in single-cell data are often multimodal, reflecting the presence of distinct subpopulations or dynamic cellular states within a heterogeneous sample. Traditional DEG methods typically rely on strong parametric assumptions, such as unimodal or normally distributed expression levels, that are rarely valid in real single-cell datasets. These assumptions can lead to biased or misleading results, especially when the underlying expression patterns deviate significantly from the assumed models. Consequently, such methods may miss subtle but important changes in gene expression or incorrectly identify genes as differentially expressed due to poor model fit.

In addition to these statistical challenges, most DEG detection tools do not adequately account for intrinsic dependencies and similarities between cells, such as those arising from shared lineage, cell cycle stage, or microenvironmental influences. Ignoring these relationships can further reduce sensitivity and increase false discovery rates. The combination of technical noise, biological variability, sparsity, and complex expression landscapes underscores the need for more flexible and robust approaches that can better capture the true structure of single-cell data while maintaining statistical rigor in DEG detection.

1.1.2 DEG detection at the pseudobulk level

Pseudobulk approaches, which aggregate expression counts across cells within a group, offer improved statistical power and reduced noise but introduce their own set of challenges. These methods similarly fall short in handling complex distributional structures and often involve tedious and multi-step preprocessing workflows. More importantly, they disregard cellular heterogeneity by collapsing diverse cell states into bulk-like averages, potentially masking cell type–specific expression changes. Furthermore, once data are aggregated, it becomes difficult to trace differential signals back to individual cells or subpopulations, limiting biological interpretability at the single-cell level.

1.2 Optimal transport in single-cell biology

Optimal Transport (OT) theory has emerged as a powerful mathematical framework for addressing a wide range of challenges in single-cell data analysis. In the domain of multi-omics data integration, recent methods such as scConfluence (Samaran et al., 2024) have employed autoencoders combined with regularized inverse OT to align weakly connected features across modalities, optimizing cell correspondence in a shared latent space via learned regularization parameters. Similarly, uniPort (Cao *et al.,* 2022) leverages a variational autoencoder (VAE) with minibatch unbalanced OT to significantly enhance computational efficiency when processing large-scale datasets. In the context of cell-type perturbation, CellOT (Bunne *et al.,* 2023) introduces a parameterized OT map, using neural networks to learn feature representations and transport parameters, while CINEMA-OT (Dong *et al.,* 2023) integrates Sinkhorn iterations with causal inference to account for confounding variables in perturbation-response modeling.

In trajectory inference, Waddington-OT (Schiebinger *et al.,* 2019) applies unbalanced OT with Sinkhorn iterations to reconstruct developmental trajectories and infer ancestor-descendant

relationships over time. Furthermore, OT has been employed in cell–cell communication modeling, as exemplified by COMMOT (Cang *et al.,* 2023), which uses high-dimensional Sinkhorn-based transport to quantify intercellular signaling patterns. OT-based methods have also been applied to differential expression (DE) analysis. For example, EMDomics (Nabavi *et al.,* 2016) utilizes Wasserstein distance with permutation testing to assess DE, and SigEMD (Wang *et al.,* 2018) incorporates zero-inflated modeling to mitigate sparsity prior to OT-based comparison. Collectively, these studies demonstrate that OT-based approaches are capable of addressing diverse analytical tasks in single-cell biology, with successful applications across various omics technologies, including scATAC-seq, spatial transcriptomics, and single-cell proteomics. Moreover, OT has been increasingly integrated with deep learning architectures such as autoencoders, VAEs, and neural networks, further enhancing its scalability and flexibility. Nonetheless, there remains a noticeable underrepresentation of OT-based methods specifically tailored for differential expression analysis at the single-cell level, highlighting an important opportunity for future methodological innovation.

2 Theoretical background

The p-Wasserstein Distance: A Metric for Comparing Distributions

The p-Wasserstein distance provides a way to measure how different two probability distributions are, particularly when they are supported over geometric spaces $\mathbb{R}^d$. It is widely used in optimal transport theory, where the goal is to find the most efficient way of transforming one distribution into another.

Mathematical Deduction

Given two probability distributions P and Q, the p-Wasserstein distance measures the minimum cost required to transport the probability mass of P to match the distribution Q. This cost is determined by the distance over which the mass is moved—raised to the power of p—and the geometry of the underlying space. Thus, the metric incorporates both spatial structure and the parameter p into the measurement of distributional dissimilarity.

Let P and Q be two probability measures in $\mathcal{P}_p(\mathbb{R}^d)$, the space of Borel probability measures on $\mathbb{R}^d$ with finite p-th moments:

$$\int_{\mathbb{R}^d} \|x\|^p \, dP(x) < \infty, \quad \int_{\mathbb{R}^d} \|y\|^p \, dQ(y) < \infty.$$

Coupling

A coupling π is a joint distribution over pairs (x, y) such that the marginal of x follows P and the marginal of y follows Q. Formally:

$$\pi \in \Pi(P, Q) \iff \pi_X = P \quad \text{and} \quad \pi_Y = Q.$$

Each π represents a transport plan, a way to move mass from P to Q.

Cost Function

The cost of moving a unit of mass from x to y is given by:

$$c(x, y) = \|x - y\|^p,$$

where $\|\cdot\|$ denotes the Euclidean norm, and $p \geq 1$ is a parameter that determines the degree to which longer transport distances are penalized. The choice of p influences the

geometry of the optimal transport problem, with higher values of p placing greater emphasis on minimizing long-range movements.

The p-Wasserstein distance is defined as the minimum expected cost over all valid couplings:

$$W_p(P, Q) = \left(\inf_{\pi \in \Pi(P,Q)} \int_{\mathbb{R}^d \times \mathbb{R}^d} \|x - y\|^p \, d\pi(x, y) \right)^{1/p}.$$

The Wasserstein distance possesses several desirable mathematical and practical properties. First, it inherently respects the geometric structure of the underlying space, as it accounts for the actual spatial arrangement of probability mass. Second, it defines a proper metric on the space of probability distributions, satisfying the standard axioms of symmetry, non-negativity, triangle inequality, and identity of indiscernibles, that is, the distance is zero if and only if the two distributions are identical. Finally, the Wasserstein distance exhibits robustness to small perturbations in the distributions, which makes it particularly well-suited for applications in domains such as image analysis, generative modelling, and computational biology.

The Sinkhorn Algorithm: Fast Approximate Optimal Transport

However, computing the exact Wasserstein distance can be computationally expensive, especially for large-scale or high-dimensional problems. The Sinkhorn algorithm provides an efficient approximation by introducing an entropy regularization term to the objective. A detailed comparison between Wasserstein distance and Sinkhorn algorithm is given below.

Table 1: Comparison between Wasserstein Distance and Sinkhorn algorithm

**Feature**	**Wasserstein Distance**	**Sinkhorn Algorithm**
Purpose	Computes the exact OT solution	Computes an approximate OT solution with entropy regularization
Formulation	Minimizes transport cost	Minimizes transport cost and entropy regularization
Regularization	None	Adds entropy term for smoothness and tractability
Complexity	High ($\mathcal{O}(n^3 \log n)$)	Faster ($\mathcal{O}(n^2)$) due to matrix operations
Scalability	Challenging for very large problems	Scalable to larger distributions
Accuracy	Exact solution	Approximate solution, depending on regularization parameter λ
Applications	Theoretical analysis, small datasets	Large-scale machine learning tasks, domain adaptation, generative models

Entropy-Regularized Transport Problem

Let $P = (p_1, \ldots, p_n)$ and $Q = (q_1, \ldots, q_m)$ be discrete probability distributions. Let $C \in \mathbb{R}^{n\times m}$ be the cost matrix, where $C_{ij} = \|x_i - y_j\|^2$. The regularized problem becomes:

$$\min_{\pi\in\mathbb{R}^{n\times m}} \sum_{i,j} \pi_{ij} C_{ij} + \lambda \sum_{i,j} \pi_{ij} \log \pi_{ij}$$

subject to:

$$\sum_j \pi_{ij} = p_i, \quad \sum_i \pi_{ij} = q_j$$

Here, $\lambda > 0$ is a regularization parameter that promotes smoothness (higher entropy) in the transport plan.

The optimal transport plan π^* has a special form:

$$\pi^* = \text{diag}(u) K \,\text{diag}(v)$$

where:

$$K = \exp\left(-\frac{C}{\lambda}\right)$$

and u and v are scaling vectors adjusted to satisfy the marginal constraints.

Iterative Scaling Procedure

The vectors $u \in \mathbb{R}^n$ and $v \in \mathbb{R}^m$ serve as multiplicative scaling factors that iteratively adjust the rows and columns of the transport plan to ensure that the marginal constraints are satisfied — that is, the resulting transport plan has marginals exactly equal to the input distributions P and Q. The Sinkhorn algorithm proceeds as follows:

1. Initialize the scaling vectors as $u^{(0)} = \mathbf{1}_n$ and $v^{(0)} = \mathbf{1}_m$, where $\mathbf{1}_n$ and $\mathbf{1}_m$ are vectors of ones of length n and m, respectively.
2. At each iteration k, update the scaling vectors according to:

$$u^{(k+1)} = \frac{P}{Kv^{(k)}}, \quad v^{(k+1)} = \frac{Q}{K^\top u^{(k+1)}}$$

   where the division is performed elementwise, and $K = \exp(-C/\lambda)$ is the Gibbs kernel derived from the cost matrix C and regularization parameter λ.
3. Iterate until convergence, typically measured by the change in the scaling vectors or by the deviation of the current marginals from the target distributions.

The Sinkhorn algorithm is particularly advantageous due to its efficiency and robustness. It significantly reduces the computational burden compared to solving the unregularized optimal transport problem, making it much faster in practice. Its scalability enables it to handle large datasets, often involving thousands of points, with ease. Moreover, the inclusion of the entropy regularization term promotes smoother and more stable transport plans by discouraging overly sharp or deterministic solutions. This balance between computational speed, scalability, and numerical stability makes Sinkhorn a practical choice for many real-world applications.

Effect of Regularization Parameter λ

The regularization parameter λ plays a crucial role in controlling the trade-off between accuracy and computational efficiency. When λ is small, the solution closely approximates the classical optimal transport plan, offering higher accuracy but at the cost of slower convergence and greater computational effort. In contrast, a larger λ leads to faster and smoother solutions by encouraging higher entropy in the transport plan, though this comes with a loss in precision. Choosing an appropriate value of λ depends on the specific application and the desired balance between fidelity and speed.

In summary, the Sinkhorn algorithm provides a fast and practical way to approximate optimal transport distances, especially useful in computer science, biology, and medicine where large datasets are common.

Kernel Density Estimation with Gaussian Kernels

In order to better capture the marginal distribution of single cell data, we applied Gaussian Kernel Density Estimation (KDE) on each group's principal component space to model the distribution of the transformed data. KDE is a widely used non-parametric technique for estimating the probability density function (PDF) of a continuous random variable from a finite set of observed data points. Unlike parametric methods, KDE does not assume a specific functional form for the underlying distribution, making it particularly useful in exploratory data analysis, especially when the true distribution is unknown or complex.

Definition

Let $\{x_1, x_2, \ldots, x_n\}$ be a set of n independent and identically distributed (i.i.d.) observations drawn from an unknown distribution with an underlying density function $f(x)$. The kernel density estimator $\hat{f}(x)$ at a target point $x \in \mathbb{R}$ is defined as:

$$\hat{f}(x) = \frac{1}{nh} \sum_{i=1}^{n} K\left(\frac{x - x_i}{h}\right)$$

where:

- $K : \mathbb{R} \to \mathbb{R}_{\geq 0}$ is a kernel function that satisfies $\int_{-\infty}^{\infty} K(u)\, du = 1$,
- $h > 0$ is the bandwidth (also known as the smoothing parameter),
- x_i are the observed data points, and
- $\hat{f}(x)$ is the estimated density at point x.

The kernel function $K(u)$ determines the shape of the "bump" placed at each data point, while the bandwidth h controls the width of these bumps. The overall estimate is obtained by summing contributions from all data points.

Gaussian Kernel

A common and effective choice for the kernel function is the Gaussian (or normal) kernel, defined as:

$$K(u) = \frac{1}{\sqrt{2\pi}} \exp\left(-\frac{1}{2}u^2\right)$$

Substituting the Gaussian kernel into the general KDE formula yields:

$$\hat{f}(x) = \frac{1}{nh}\sum_{i=1}^{n} \frac{1}{\sqrt{2\pi}} \exp\left(-\frac{1}{2}\left(\frac{x - x_i}{h}\right)^2\right)$$

This can be further simplified to:

$$\hat{f}(x) = \frac{1}{nh\sqrt{2\pi}}\sum_{i=1}^{n} \exp\left(-\frac{(x - x_i)^2}{2h^2}\right)$$

which represents a smooth, continuous estimate of the underlying density obtained by summing Gaussian-shaped kernels centered at each data point.

Bandwidth Selection

The choice of bandwidth h plays a pivotal role in the performance of kernel density estimation. A small bandwidth produces a highly detailed density estimate that may closely follow the empirical data. While this can reveal fine-scale structures, it also increases the risk of overfitting, resulting in a noisy and overly jagged density curve that captures spurious fluctuations rather than meaningful patterns. Conversely, a large bandwidth generates a much smoother estimate by averaging over broader regions of the data space. Although this can reduce variance and noise, it may also lead to underfitting, potentially masking important features of the underlying distribution such as multimodality, asymmetry, or sharp peaks.

Several methods exist for selecting an appropriate bandwidth, ranging from cross-validation to plug-in estimators. In this work, we adopt a widely used rule-of-thumb known as Scott's Rule, which provides a closed-form expression for the bandwidth under the assumption of normally distributed data:

$$h = 3.5\,\sigma\,n^{-1/3}$$

where σ is the empirical standard deviation of the dataset and n is the number of samples. Scott's Rule balances the trade-off between bias and variance in the density estimate and is particularly effective for unimodal, approximately Gaussian data.

Wald Test

After estimating the transported expression matrix, we calculated per-gene differences by subtracting the original expression values from the transported ones. The mean of these differences across cells was then computed to quantify the overall directional shift in gene expression, and the corresponding standard error was estimated to assess variability. To evaluate statistical significance, we applied the Wald test by comparing each gene's mean difference against zero, under the null hypothesis that there is no expression difference between the two groups. The Wald statistic assesses whether a parameter estimate, relative to its standard error, significantly deviates from a specified value, and is widely used in both regression analysis and general hypothesis testing frameworks. The Wald test statistic is calculated as:

$$W = \frac{(\hat{\theta})^2}{\mathrm{Var}(\hat{\theta})}$$

where:

- $\hat{\theta}$ is the estimated parameter.
- $\mathrm{Var}(\hat{\theta})$ is the variance of the estimated parameter.

3 OTMODE pseudocode

Algorithm 1 Detecting Differential Features Between Conditions

Input: Multi-omics matrix, threshold α
Output: Differentially expressed features
$C_1 \leftarrow$ Condition 1 cells, $C_2 \leftarrow$ Condition 2 cells
$\rho_1 \leftarrow \text{Density}(C_1)$, $\rho_2 \leftarrow \text{Density}(C_2)$
$D \leftarrow \text{EuclideanDistanceMatrix}(C_1, C_2)$
$\Gamma \leftarrow \text{UnbalancedSinkhorn}(\rho_1, \rho_2, D)$
$C_1' \leftarrow \Gamma(C_2)$
for each feature f **do**
    $p_f \leftarrow \text{WaldTest}(C_1[f], C_1'[f])$
    if $p_f < \alpha$ **then**
        Mark f as significant
    end if
end for
return List of significant features

Algorithm 2 Enhancing Cell-Type Annotation via Optimal Transport

Input: Data matrix, marker genes, threshold α
Output: Annotated clusters
$\mathcal{C} \leftarrow$ Clustered cells
$\mathcal{C}' \leftarrow \{C_i \in \mathcal{C} \mid \text{prop}(C_i) \geq \tau\}$
for each $(C_i, C_j) \in \mathcal{C}' \times \mathcal{C}'$ **do**
    $\text{OTD}_{ij} \leftarrow \text{UnbalancedSinkhornDistance}(C_i, C_j, \text{marker\_genes})$
end for
for each $C_i \in \mathcal{C}'$, cell type T **do**
    $p_i^T \leftarrow \text{PermutationTest}(C_i, T, \text{OTD})$
    if C_i has highest OTD for T and $p_i^T < \alpha$ **then**
        Assign $C_i \rightarrow T$
    else
        Label C_i as "unknown"
    end if
end for
return Annotated clusters

4 Supplementary figures

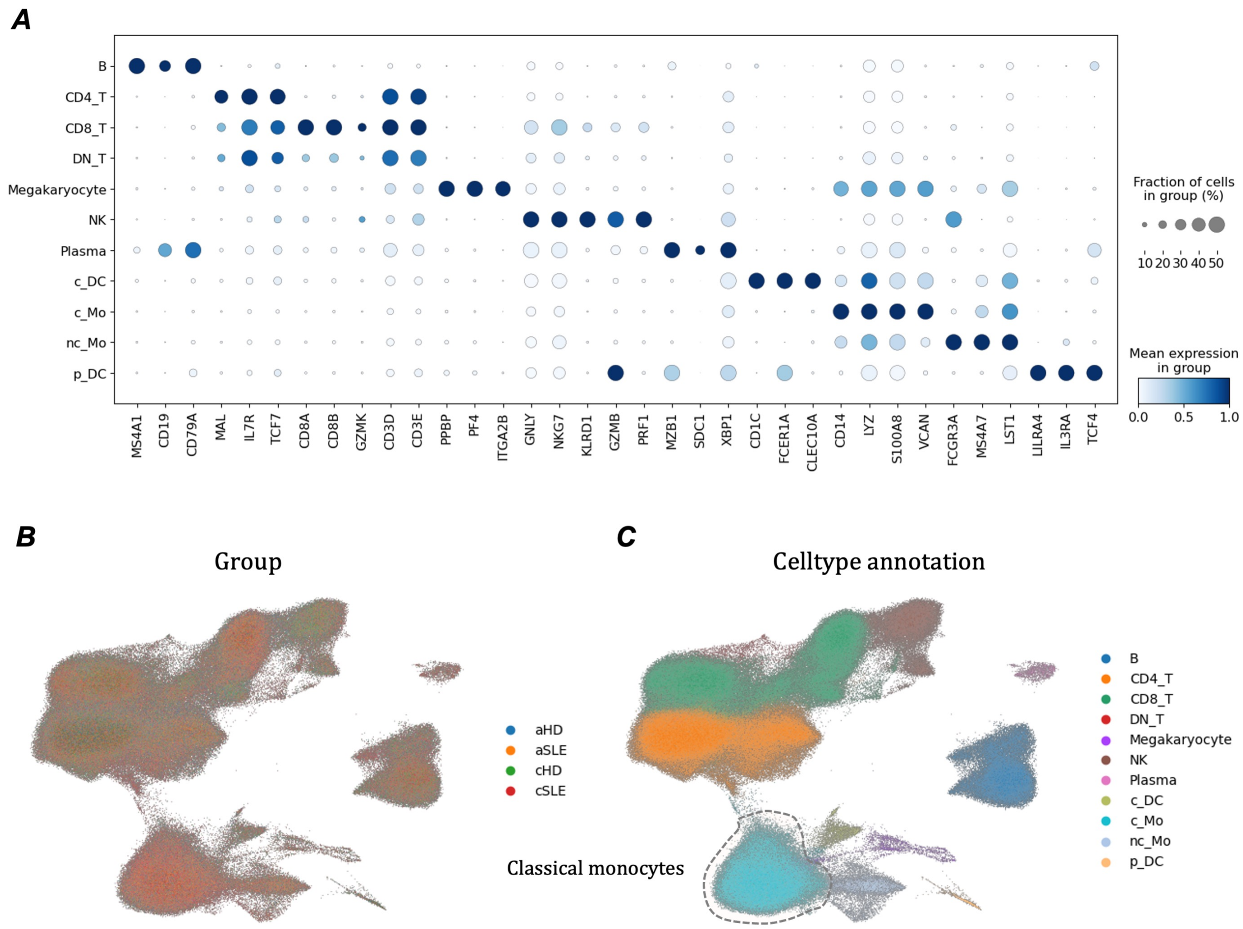
A
B
CD4_T
CD8_T
DN_T
Megakaryocyte
NK
Plasma
c_DC
c_Mo
nc_Mo
p_DC
MS4A1
CD19
CD79A
MAL
IL7R
TCF7
CD8A
CD8B
GZMK
CD3D
CD3E
PPBP
PF4
ITGA2B
GNLY
NKG7
KLRD1
GZMB
PRF1
MZB1
SDC1
XBP1
CD1C
FCER1A
CLEC10A
CD14
LYZ
S100A8
VCAN
FCGR3A
MS4A7
LST1
LILRA4
IL3RA
TCF4
Fraction of cells in group (%)
10 20 30 40 50
Mean expression in group
0.0 0.5 1.0
B
Group
aHD
aSLE
cHD
cSLE
C
Celltype annotation
Classical monocytes
B
CD4_T
CD8_T
DN_T
Megakaryocyte
NK
Plasma
c_DC
c_Mo
nc_Mo
p_DC

Supp figure 1: UMAP visualization of SLE scRNA-seq data.

A. Dotplot for immune cell marker genes. Color scales indicate normalized gene expression per celltype.

B. UMAP visualization of group information where different cohorts overlap. aHD: healthy adults; aSLE: adults with SLE; cHD: healthy children; cSLE: children with SLE.

C. Cell-type annotation on UMAP plot, with classical monocytes highlighted. c_MO, classical monocytes; nc_MO, non-classical monocytes; DN_T, double-negative T cells; c_DC, conventional dendritic cells; pDC, plasmacytoid dendritic cells.

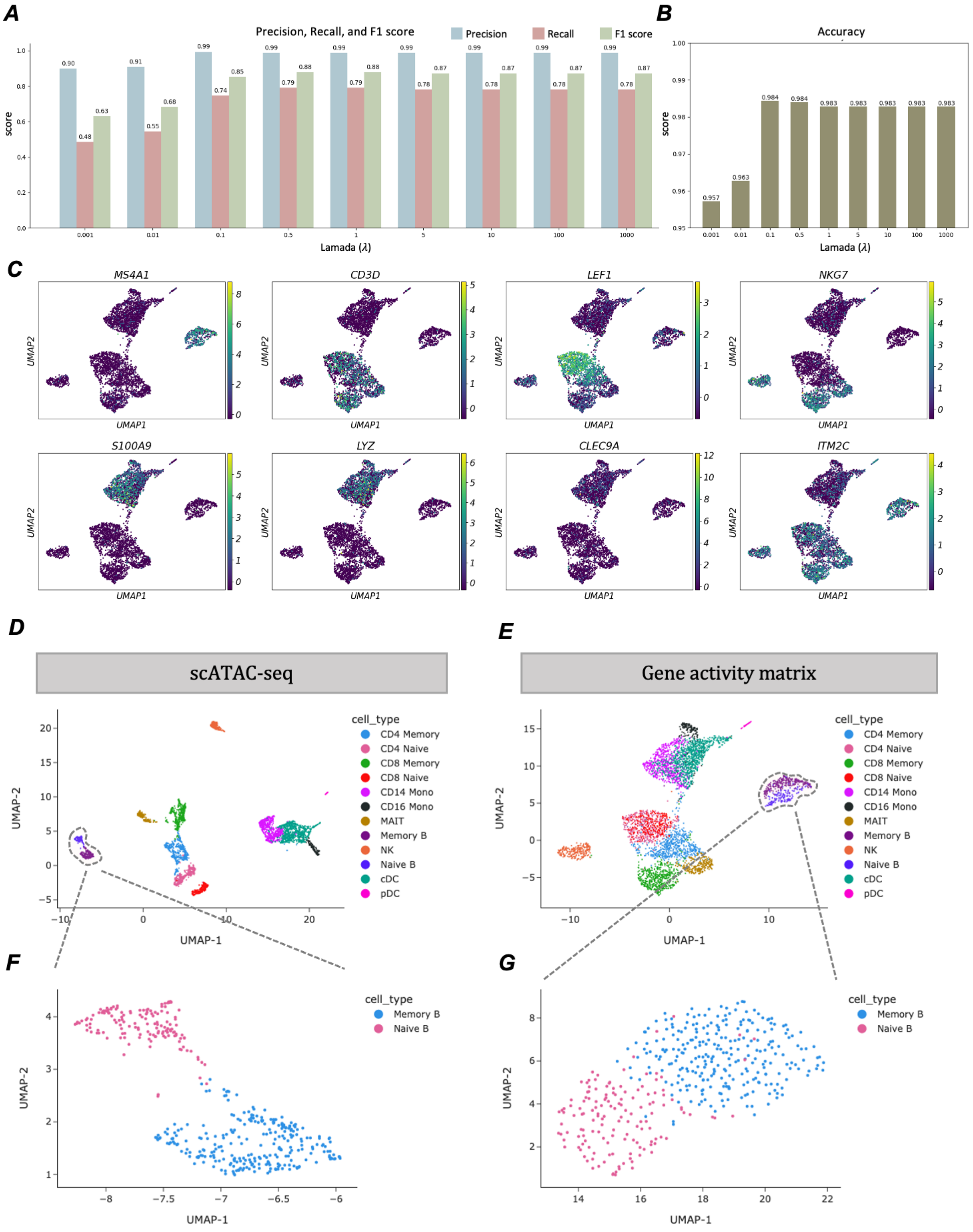
A
Precision, Recall, and F1 score
Precision
Recall
F1 score
score
Lamada (λ)
B
Accuracy
score
Lamada (λ)
C
MS4A1
CD3D
LEF1
NKG7
S100A9
LYZ
CLEC9A
ITM2C
UMAP1
UMAP2
D
scATAC-seq
E
Gene activity matrix
cell_type
CD4 Memory
CD4 Naive
CD8 Memory
CD8 Naive
CD14 Mono
CD16 Mono
MAIT
Memory B
NK
Naive B
cDC
pDC
UMAP-1
UMAP-2
F
G
Memory B
Naive B

Supp figure 2: OTMODE regularization strength (λ) benchmarking and UMAP visualization of healthy human PBMC using scATAC-seq accessibility and derived gene activity.

A. Barplot for comparing precision, recall, and F1 score under different regularization strengths in simulated scATAC-seq data.

B. Barplot for accuracy comparison under different regularization strengths in simulated scATAC-seq data, where regularization strength λ is in x-axis.

C. UMAP embeddings of single-cell accessibility scores for eight canonical marker genes: MS4A1 (B cells), CD3D (T cells), LEF1 (naïve T cells), NKG7 (NK cells), S100A9 (monocytes), LYZ (monocytes), CLEC9A (cDC), and ITM2C (pDC). Color scales indicate normalized chromatin-derived gene expression.

D. UMAP embedding of the normalised scATAC-seq profiles, with cells colored by manually curated cell-type annotation (CD4 Memory, CD4 Naïve, CD8 Memory, CD8 Naïve, CD14 Mono, CD16 Mono, MAIT, Memory B, NK, Naïve B, cDC, pDC).

E. UMAP embedding of the gene activity matrix (chromatin-derived "expression"), with the same cell-type color scheme, demonstrating concordant cell-type clustering.

F. Zoom-in on the scATAC-seq UMAP highlighting Memory B versus Naïve B populations.

G. Zoom-in on the gene activity UMAP highlighting the same Memory B and Naïve B populations.

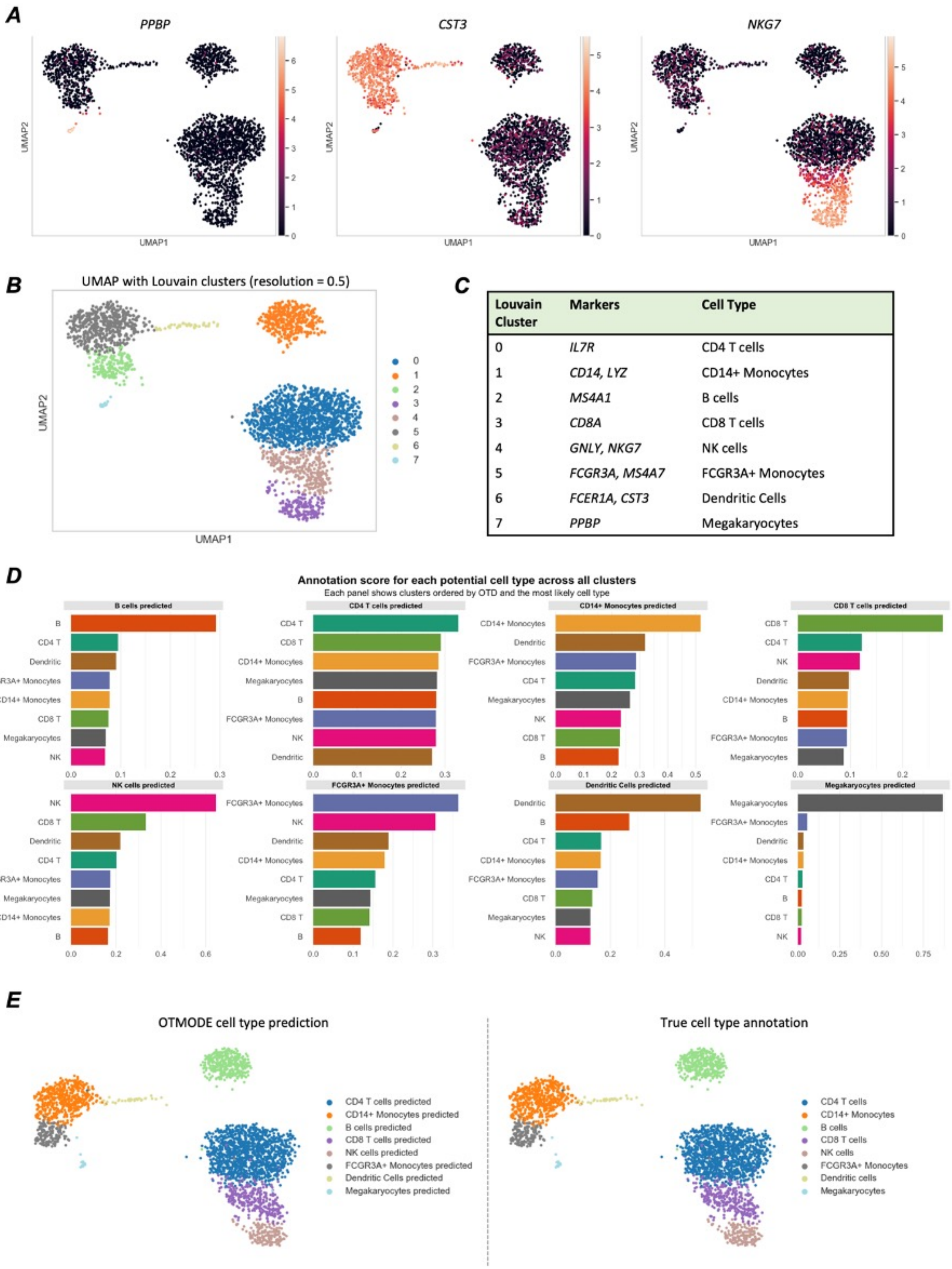
A
PPBP
CST3
NKG7
UMAP1
UMAP2
B
UMAP with Louvain clusters (resolution = 0.5)
C
Louvain Cluster | Markers | Cell Type
0 | IL7R | CD4 T cells
1 | CD14, LYZ | CD14+ Monocytes
2 | MS4A1 | B cells
3 | CD8A | CD8 T cells
4 | GNLY, NKG7 | NK cells
5 | FCGR3A, MS4A7 | FCGR3A+ Monocytes
6 | FCER1A, CST3 | Dendritic Cells
7 | PPBP | Megakaryocytes
D
Annotation score for each potential cell type across all clusters
Each panel shows clusters ordered by OTD and the most likely cell type
B cells predicted
CD4 T cells predicted
CD14+ Monocytes predicted
CD8 T cells predicted
NK cells predicted
FCGR3A+ Monocytes predicted
Dendritic Cells predicted
Megakaryocytes predicted
E
OTMODE cell type prediction
CD4 T cells predicted
CD14+ Monocytes predicted
B cells predicted
CD8 T cells predicted
NK cells predicted
FCGR3A+ Monocytes predicted
Dendritic Cells predicted
Megakaryocytes predicted
True cell type annotation
CD4 T cells
CD14+ Monocytes
B cells
CD8 T cells
NK cells
FCGR3A+ Monocytes
Dendritic cells
Megakaryocytes

Supp figure 3: OTMODE facilitates cell-type annotation by measuring the mover's distances of multiple marker genes across clusters.

A. Feature plots for three immune cell marker genes on UMAP coordinates.

B. Selected marker genes for each cell-type and corresponding cluster identified by Louvain clustering. Cell-type information is provided in the data.

C. Louvain clusters on UMAP plot under the resolution of 0.5.

D. Barplot to show the mover's distance (y-axis) in each cluster (i.e., predicted cell-type in x-axis) using marker genes of each cell-type listed above. Different colors indicate marker genes from cell-types. The highest bar for each cluster was further assigned the corresponding cell-type.

E. UMAP plots to illustrate the comparison between the OTMODE predicted cell-types (left panel) and the true cell-type annotation (right panel).

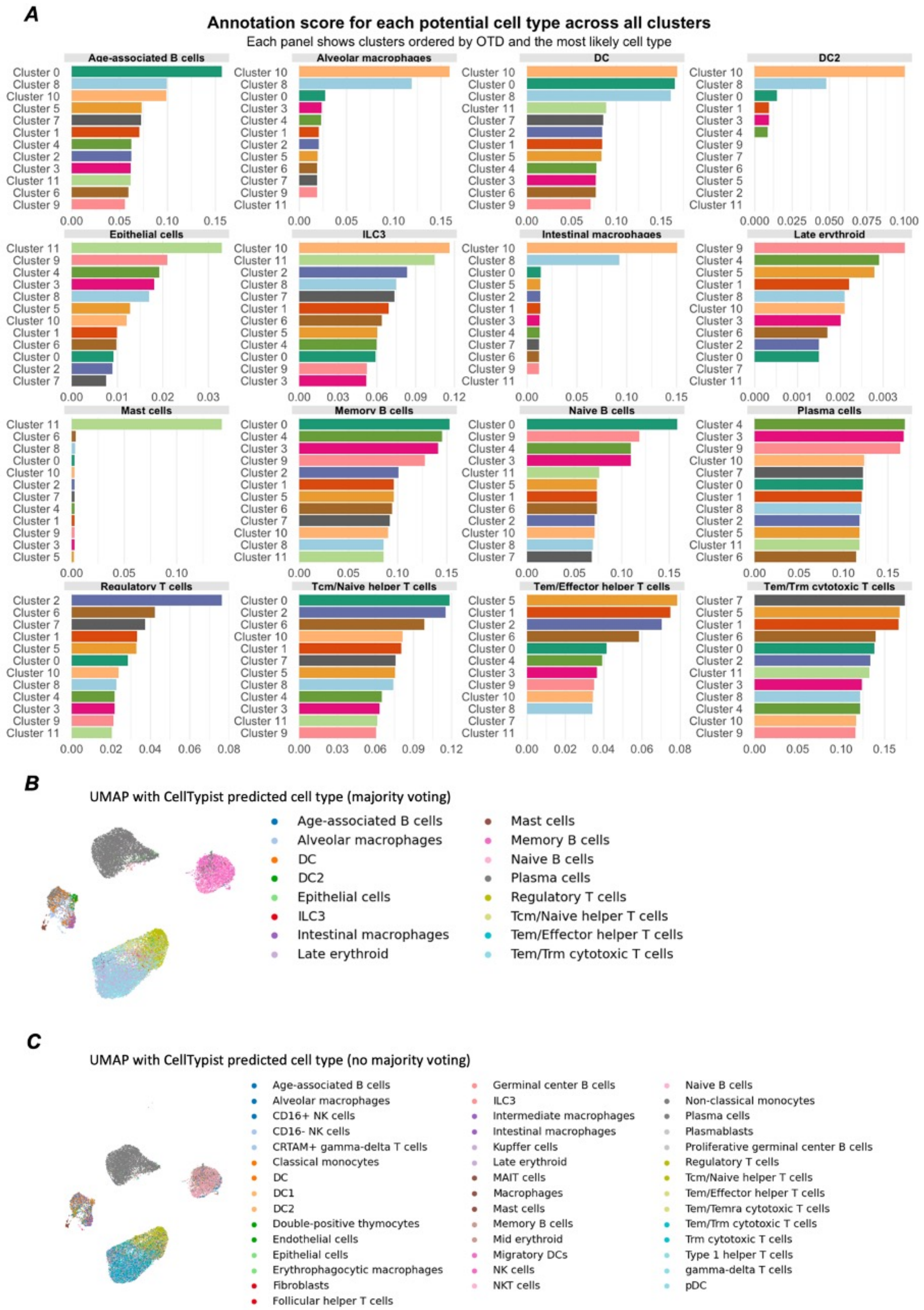
A
Annotation score for each potential cell type across all clusters
Each panel shows clusters ordered by OTD and the most likely cell type
Age-associated B cells
Alveolar macrophages
DC
DC2
Epithelial cells
ILC3
Intestinal macrophages
Late erythroid
Mast cells
Memory B cells
Naive B cells
Plasma cells
Regulatory T cells
Tcm/Naive helper T cells
Tem/Effector helper T cells
Tem/Trm cytotoxic T cells
B
UMAP with CellTypist predicted cell type (majority voting)
Age-associated B cells
Alveolar macrophages
DC
DC2
Epithelial cells
ILC3
Intestinal macrophages
Late erythroid
Mast cells
Memory B cells
Naive B cells
Plasma cells
Regulatory T cells
Tcm/Naive helper T cells
Tem/Effector helper T cells
Tem/Trm cytotoxic T cells
C
UMAP with CellTypist predicted cell type (no majority voting)
Age-associated B cells
Alveolar macrophages
CD16+ NK cells
CD16- NK cells
CRTAM+ gamma-delta T cells
Classical monocytes
DC
DC1
DC2
Double-positive thymocytes
Endothelial cells
Epithelial cells
Erythrophagocytic macrophages
Fibroblasts
Follicular helper T cells
Germinal center B cells
ILC3
Intermediate macrophages
Intestinal macrophages
Kupffer cells
Late erythroid
MAIT cells
Macrophages
Mast cells
Memory B cells
Mid erythroid
Migratory DCs
NK cells
NKT cells
Naive B cells
Non-classical monocytes
Plasma cells
Plasmablasts
Proliferative germinal center B cells
Regulatory T cells
Tcm/Naive helper T cells
Tem/Effector helper T cells
Tem/Temra cytotoxic T cells
Tem/Trm cytotoxic T cells
Trm cytotoxic T cells
Type 1 helper T cells
gamma-delta T cells
pDC

Supp figure 4: Evaluation of CellTypist annotation accuracy by OTMODE.

A. Annotation scores of each cluster for potential cell types based on OTD. Each panel corresponds to a predicted cell type, with bars representing clusters sorted by their OTD score to that cell type. Higher OTDs suggest greater alignment to the indicated cell type. The x-axis denotes the computed OTD score, and the y-axis lists the clusters.

B. UMAP projection of single cells colored by CellTypist-predicted labels using majority voting. Each cell is assigned to a cell type based on the most frequent CellTypist prediction within its local neighborhood.

C. UMAP projection of single cells colored by full CellTypist prediction (without majority voting). ILC3, Type 3 innate lymphoid cells; DC1, Type 1 dendritic cells; DC2, Type 2 dendritic cells; pDC, Plasmacytoid dendritic cells; MAIT, Mucosal-associated invariant T cells.

References

[1] Handley, A., et al. (2015). Designing cell-type-specific genome-wide experiments. *Molecular Cell*, *58*(4), 621–631.

[2] Burkhardt, D. B., et al. (2021). Quantifying the effect of experimental perturbations at single-cell resolution. *Nature Biotechnology*, *39*(5), 619–629.

[3] Samaran, J., Peyré, G., & Cantini, L. (2024). scConfluence: single-cell diagonal integration with regularized Inverse Optimal Transport on weakly connected features. *Nature Communications*, *15*, 7762.

[4] Cao, K., Gong, Q., Hong, Y., & Wan, L. (2022). A unified computational framework for single-cell data integration with optimal transport. *Nature Communications*, *13*, 7419.

[5] Bunne, C., Stark, S. G., Gut, G., et al. (2023). Learning single-cell perturbation responses using neural optimal transport. *Nature Methods*, *20*(11), 1759–1768.

[6] Dong, M., Wang, B., Wei, J., et al. (2023). Causal identification of single-cell experimental perturbation effects with CINEMA-OT. *Nature Methods*, *20*(11), 1769–1779.

[7] Nabavi, S., Schmolze, D., Maitituoheti, M., Malladi, S., & Beck, A. H. (2016). EMDomics: a robust and powerful method for the identification of genes differentially expressed between heterogeneous classes. *Bioinformatics*, *32*(4), 533–541.

[8] Wang, T., & Nabavi, S. (2018). SigEMD: A powerful method for differential gene expression analysis in single-cell RNA sequencing data. *Methods*, *145*, 25–32.

[9] Schiebinger, G., Shu, J., Tabaka, M., et al. (2019). Optimal-Transport Analysis of Single-Cell Gene Expression Identifies Developmental Trajectories in Reprogramming. *Cell*, *176*(4), 928–943.e22.

[10] Cang, Z., Zhao, Y., Almet, A. A., et al. (2023). Screening cell–cell communication in spatial transcriptomics via collective optimal transport. *Nature Methods*, *20*(2), 218–228.
