# Supplementary figures and images for: OTMODE: an optimal transport theory-based framework for identifying differential features in single-cell multi-omics data
Source: Bioinformatics. 2025 Dec 3;42(1):btaf650. doi: 10.1093/bioinformatics/btaf650 (PMC12766913; doi:10.1093/bioinformatics/btaf650)

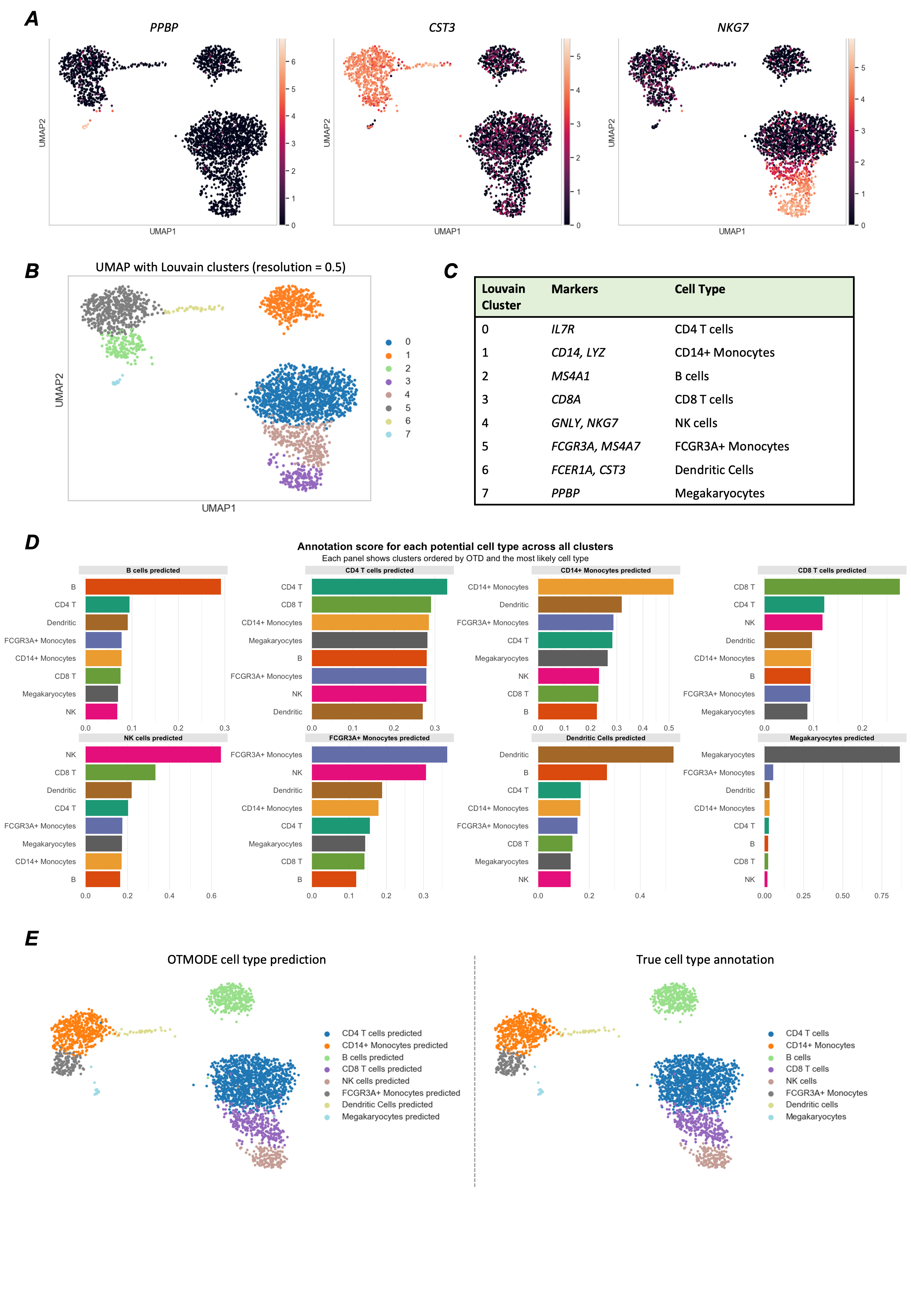

Supplement: btaf650_Supplementary_Data [file btaf650_supplementary_data.zip › Slide9.png]

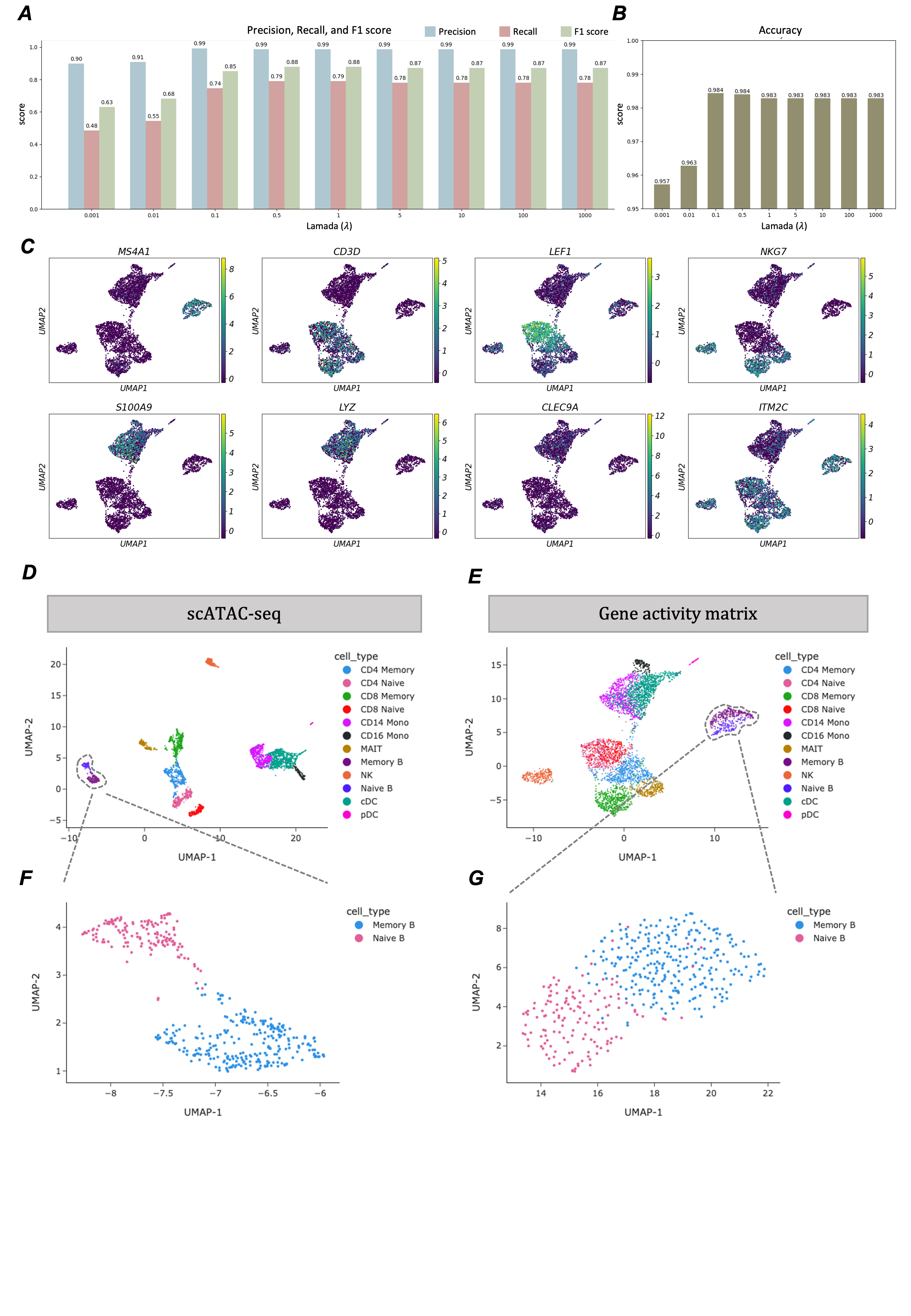

Supplement: btaf650_Supplementary_Data [file btaf650_supplementary_data.zip › Slide8.png]

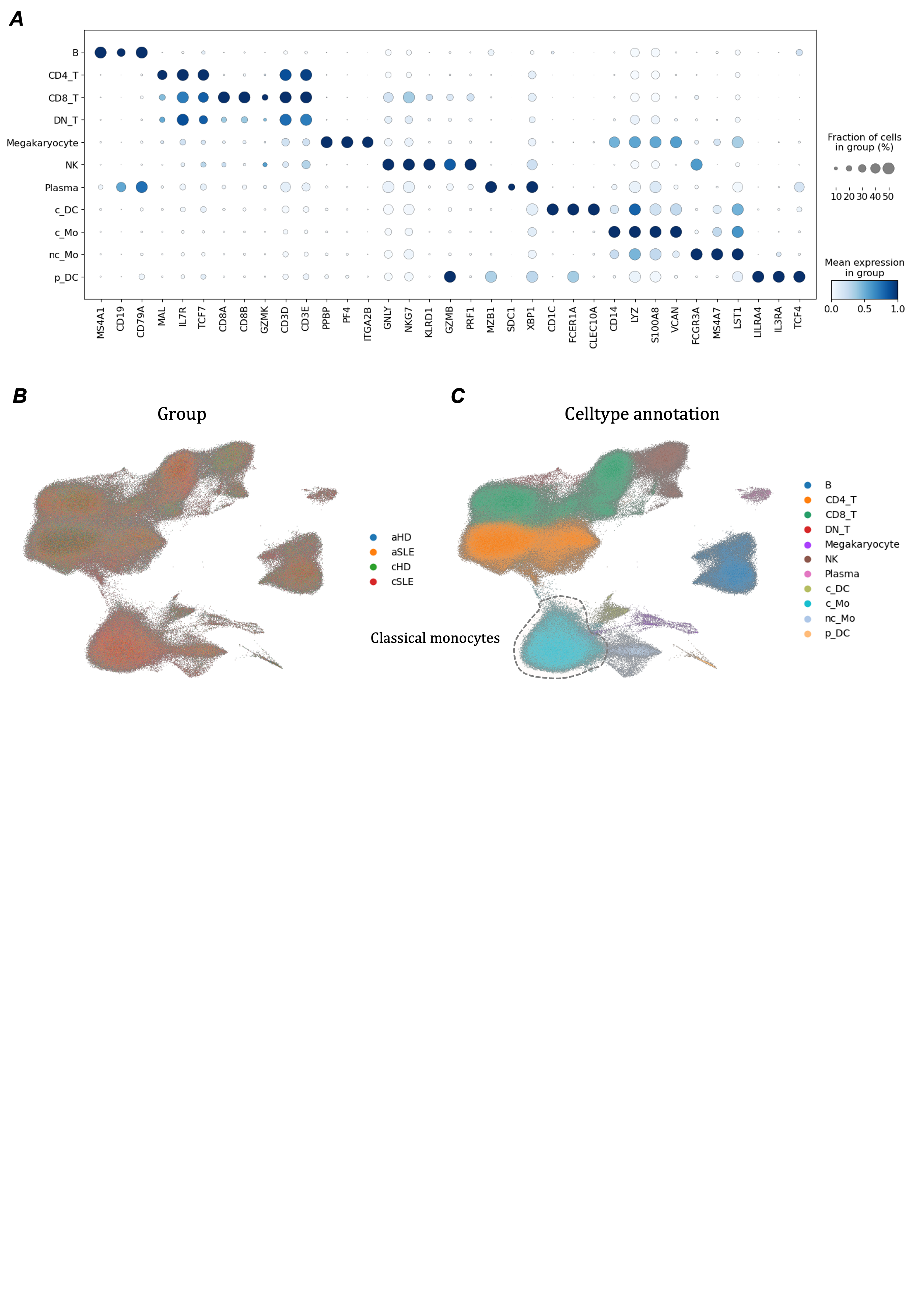

Supplement: btaf650_Supplementary_Data [file btaf650_supplementary_data.zip › Slide7.png]

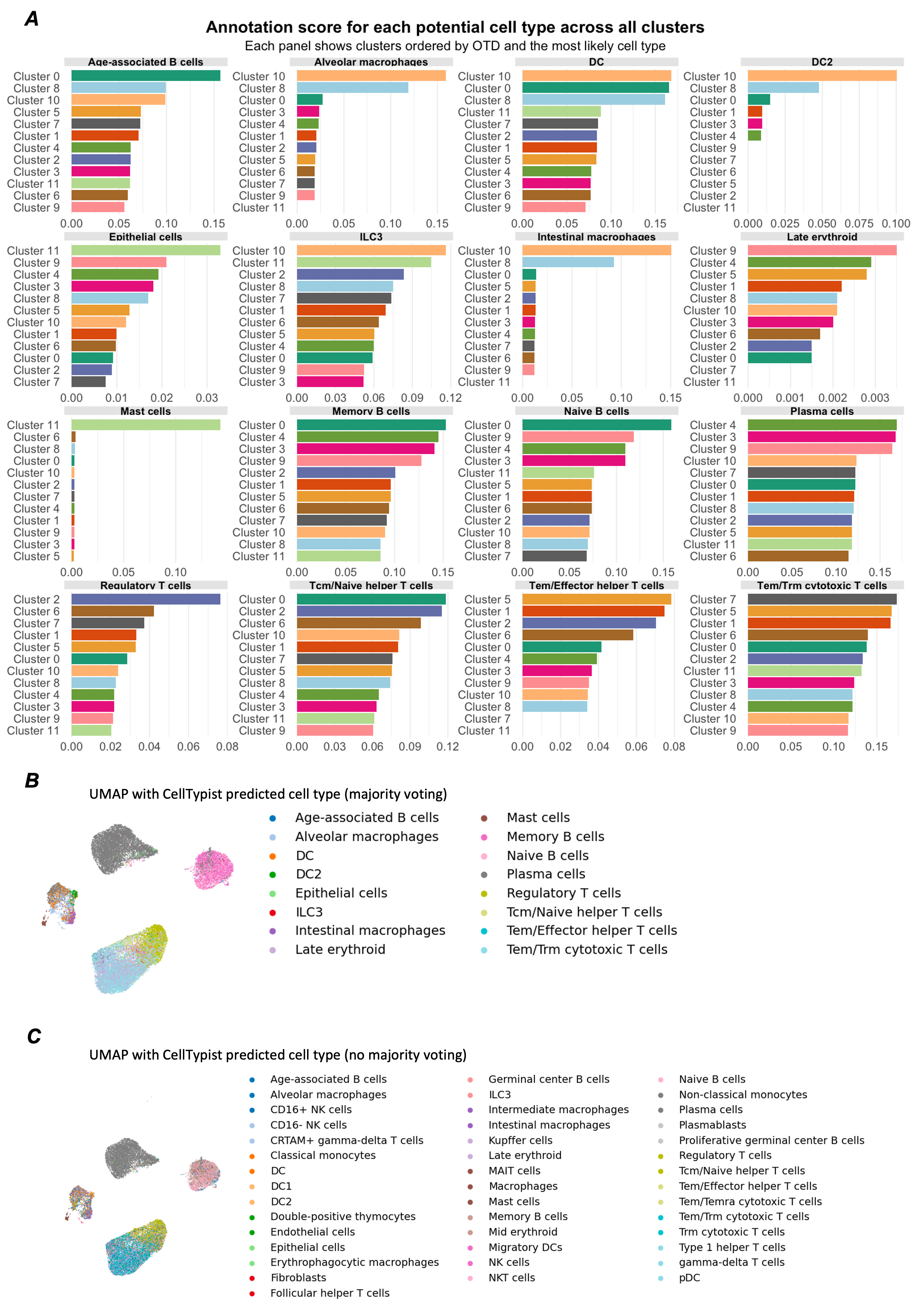

Supplement: btaf650_Supplementary_Data [file btaf650_supplementary_data.zip › Slide10.png]
